# Endogenous Modulation of Extracellular Matrix Collagen during Scar Formation after Myocardial Infarction

**DOI:** 10.3390/ijms232314571

**Published:** 2022-11-23

**Authors:** David Schumacher, Adelina Curaj, Mareike Staudt, Sakine Simsekyilmaz, Isabella Kanzler, Peter Boor, Barbara Mara Klinkhammer, Xiaofeng Li, Octavian Bucur, Adnan Kaabi, Yichen Xu, Huabo Zheng, Pakhwan Nilcham, Alexander Schuh, Mihaela Rusu, Elisa A. Liehn

**Affiliations:** 1 Department of Anesthesiology, University Hospital, RWTH Aachen University, 52074 Aachen, Germany; 2 Institute of Experimental Medicine and Systems Biology, RWTH Aachen University, 52074 Aachen, Germany; 3 Institute for Molecular Cardiovascular Research (IMCAR), RWTH Aachen University, 52074 Aachen, Germany; 4 Department for Cardiology, Angiology and Internal Intensive Care, Medical Faculty, RWTH Aachen University, 52074 Aachen, Germany; 5 Institute for Pathology, RWTH Aachen University, 52074 Aachen, Germany; 6 Division of Nephrology and Clinical Immunology, RWTH Aachen University, 52074 Aachen, Germany; 7 Institute of Molecular Biomedicine, Comenius University, 811 08 Bratislava, Slovakia; 8 “Victor Babes” National Institute of Pathology, 050096 Bucharest, Romania; 9 Viron Molecular Medicine Institute, 1 Boston Place, Ste 2600, Boston, MA 02108, USA; 10 Institute for Molecular Medicine, University of Southern Denmark, 5230 Odense, Denmark; 11 National Heart Center Singapore, 5 Hospital Dr., Singapore 169609, Singapore

**Keywords:** myocardial infarction, scar formation, extracellular matrix, remodeling, inflammation

## Abstract

Myocardial infarction is remains the leading cause of death in developed countries. Recent data show that the composition of the extracellular matrix might differ despite similar heart function and infarction sizes. Because collagen is the main component of the extracellular matrix, we hypothesized that changes in inflammatory cell recruitment influence the synthesis of different collagen subtypes in myofibroblasts, thus changing the composition of the scar. We found that neutrophils sustain the proliferation of fibroblasts, remodeling, differentiation, migration and inflammation, predominantly by IL-1 and PPARγ pathways (n = 3). They also significantly inhibit the mRNA expression of fibrillar collagen, maintaining a reduced stiffness in isolated myofibroblasts (n = 4–5). Reducing the neutrophil infiltration in CCR1^−/−^ resulted in increased mRNA expression of collagen 11, moderate expression of collagen 19 and low expression of collagen 13 and 26 in the scar 4 weeks post infarction compared with other groups (n = 3). Mononuclear cells increased the synthesis of all collagen subtypes and upregulated the NF-kB, angiotensin II and PPARδ pathways (n = 3). They increased the synthesis of collagen subtypes 1, 3, 5, 16 and 23 but reduced the expression of collagens 5 and 16 (n = 3). CCR2^−/−^ scar tissue showed higher levels of collagen 13 (n = 3), in association with a significant reduction in stiffness (n = 4–5). Upregulation of the inflammation-related genes in myofibroblasts mostly modulated the fibrillar collagen subtypes, with less effect on the FACIT, network-forming and globular subtypes (n = 3). The upregulation of proliferation and differentiation genes in myofibroblasts seemed to be associated only with the fibrillar collagen subtype, whereas angiogenesis-related genes are associated with fibrillar, network-forming and multiplexin subtypes. In conclusion, although we intend for our findings to deepen the understanding of the mechanism of healing after myocardial infarction and scar formation, the process of collagen synthesis is highly complex, and further intensive investigation is needed to put together all the missing puzzle pieces in this still incipient knowledge process.

## 1. Introduction

Myocardial infarction (MI) remains the most frequent cause of death in developed countries. Despite the considerable progress in revascularization therapies, the patients often present with permanent loss of myocardial tissue, with varying degrees of scar formation, followed by progressive worsening of heart function due to adverse remodeling. Increasing evidence highlights the role of scar tissue in supporting the mechanics and functionality of the heart [1,2].

Contrary to the chronic fibrotic processes, the formation of cardiac scar tissue after an acute myocardial infarction is a highly dynamic process and is essential in rapidly re-establishing the integrity and functional stability of tissue [3,4,5]. Shortly after the acute ischemic event, the fibroblasts are activated and differentiate into myofibroblasts, representing the main source of extracellular matrix during healing after myocardial infarction. In response to the extended tissular destruction [6], they immediately being to synthetize a provisional fibrin-based matrix [7] under the monitoring of inflammatory cells [8,9,10]. This provisional matrix is gradually replaced by a strong, mature and mechanically stable collagen matrix [11,12,13,14] in an attempt to avoid negative ventricular remodeling and preserve heart function [15,16,17,18].

Recently, an increasing number of studies have reported significant differences in heart function despite similar infarction size [19,20,21,22,23,24]. Moreover, the condition hitherto believed to be necessary for reduced collagen content in the scar for improved heart function is not supported by new experimental studies [24,25,26,27]. For example, induction of myocardial infarction in CCR2^−/−^ mice known to have reduced inflammatory monocyte recruitment [28,29] results in reduced collagen content in the scar in association with decreased infarction size and a preserved ejection fraction [28,29]. In contrast, the induction of myocardial infarction in CCR1^−/−^ and CXCR4^−/−^ mice, which have reduced neutrophil recruitment [25,26], results in an increased or constant collagen content in the infarcted areas, despite preserved heart function and decreased infarction size [25,26]. All these observations can be explained only by the varying quality of the extracellular matrix composition. Collagen is the main component of the mature scar after myocardial infarction and constitutes 28 subtypes [30]. Thus, we hypothesized that changing environmental conditions during healing after myocardial infarction, such as reduced inflammatory monocytes in the case of CCR2^−/−^ mice or reduced neutrophil infiltration in the case of CCR1^−/−^ mice, can have a significant influence on the synthesis of collagen subtypes, thus changing the composition of the scar and its mechanical properties. 

Therefore, in this study, we focus on dissecting the differential expression of collagen subtypes, as well as the role of immune cells (using isolated cells and knockout mouse models) in influencing the synthesis of collagen subtypes after myocardial infarction.

## 2. Results

### 2.1. Resident Fibroblasts Are the Main Source of Collagen after Myocardial Infarction

Myofibroblasts are the primary producers of extracellular matrix in all fibrotic diseases, including scar formation after myocardial infarction. Currently, the major fibrillar collagen 1 and collagen 3 are believed to be the principal structural components of extracellular matrix and scar tissue formation. However, knowledge of time-dependent collagen subtype expression is still lacking. Therefore, we aim to assess the expression of various collagen subtypes during the 4 weeks of healing after murine myocardial infarction, as well as the potential main source of the extracellular matrix after myocardial infarction.

**Figure 1:**
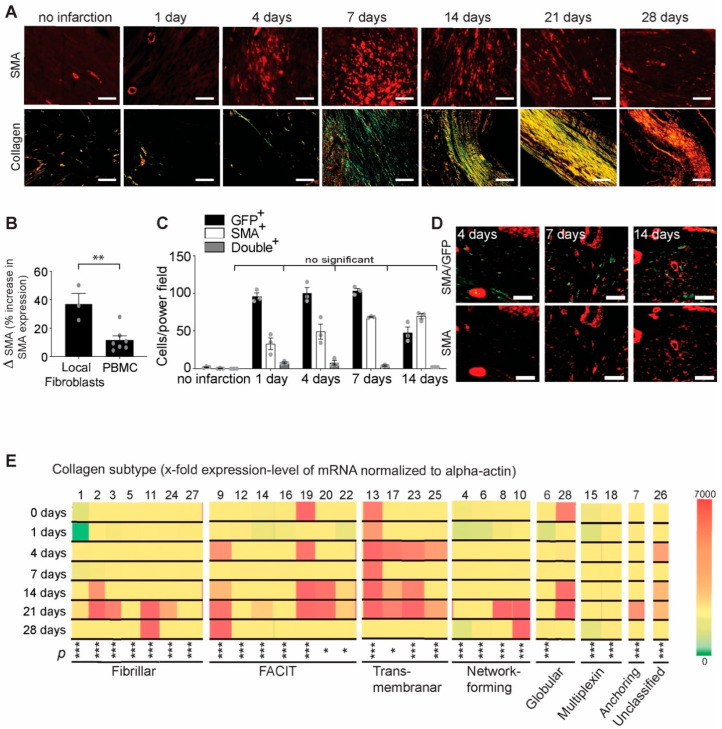
Characterization the origin and collagen subtype expression of myofibroblasts after myocardial infarction. (**A**) Time course of alpha smooth muscle actin staining (red, upper panel; scale bar, 50 µm) and Sirius red staining (lower panel, scale bar 200 µm) after myocardial infarction. (**B**) Percentage of alpha smooth muscle actin-positive (myofibroblasts) cells differentiated from cardiac fibroblasts and blood mononuclear cells (PBMC). As determined by α-SMA staining, local cardiac fibroblasts have an increased potential to differentiate towards myofibroblasts under TGF-ß1 stimulation and under hypoxic conditions compared with blood cells (** *p* < 0.01, *t*-test, n = 3–7 mice). (**C**) GFP-positive bone marrow reconstitution of lethally irradiated mice reveals that local differentiated myofibroblasts constitute most of the cells responsible for extracellular matrix synthesis; GFP/SMA double-positive cells (gray columns) are not significant compared with the control (two-way ANOVA followed by Tukey’s post hoc test, n = 3 mice per group). (**D**) Selected representative images of GFP/SMA double-positive cells (**upper panel**, yellow) and α-SMA cells (**lower panel**, red) at the time points characteristic of increased presence of myofibroblasts (scale bar, 50 µm). (**E**) mRNA expression levels of different collagen subtypes at different time points after myocardial infarction (normalized to α-actin) (* *p* < 0.05, *** *p* < 0.001, one-way ANOVA for each collagen subtype followed by Tukey’s post hoc test, n = 3 mice per group).

As expected, immunofluorescence staining for smooth muscle actin showed an increased number of differentiated myofibroblasts after myocardial infarction, peaking on day 7 and decreasing afterward (Figure 1A), corresponding to the transient activation of fibroblasts (which became transient myofibroblasts) during healing. The immunohistology images of increased smooth muscle actin (excluding the positive staining inside the vasculatures) were associated with the formation of collagen as depicted by polarized imaging of Sirius red staining. One day post infarction, there was no visible α-SMA staining and no visible collagen. The activated myofibroblasts began to significantly express α-SMA 4 days after infarction injury and peaked after 7 days (Figure 1A), when a vague green color of newly formed collagen fibers started to appear in a heterogeneous distribution. By 14 days post infarction, the differentiated myofibroblasts secreted more mature collagen fibers, probably to structurally and mechanically support the scar area. The activation of myofibroblasts is a transient process that occurs during healing after myocardial infarction and is required for maturation and stabilization of the scar [3]. At the end of the healing process, the average orientation of mature, thick collagen fibers seemed to follow an axial orientation, which progressively realigns 28 days post MI in a tide parallel organization of various dimensions.

To examine the differentiation potential of fibroblasts and their possible origins, we employed murine heart tissue and blood as myofibroblast sources and used TGF-β1 to differentiate them toward myofibroblast phenotypes. Both types of cells have the potential to differentiate; however, as expected, the resident fibroblasts presented a significantly higher potential to differentiate compared with cells recruited from blood (Figure 1B). Fate-tracing experiments of GFP-marked bone-marrow-derived cells revealed that most of the α-SMA-positive myofibroblasts were not GFP-positive during healing after myocardial infarction. The number of double-positive cells for GFP and αSMA (with possible blood origin) was not significantly increased compared with the control (with no infarction) (Figure 1C,D). This also suggested that the myofibroblasts have increased flexibility to change their synthesis in response to surrounding factors.

To assess the synthesizing function of myofibroblasts, we investigated the gene expression levels of collagen subtypes during 4 weeks of healing after myocardial infarction. We observed that during the inflammatory phase, on day 4 after myocardial infarction, transmembrane collagens and some fibril-associated collagens with interrupted mRNA gene expression of triple-helix (FACIT) collagens (collagen 9 and 19) were highly upregulated (Figure 1E). Furthermore, immediately after myocardial infarction, differentiated myofibroblasts mediated the dysregulated processes that may result in loss of tissue function via the replacement of specialized tissue by transmembrane and FACIT types of collagens (Figure 1E). Although FACIT mRNA gene expressions (collagen 9 and 19) were required in the early healing phase, on days 7 and 10 after myocardial infarction, most of the gene expression (collagen 9, 14, 19, 20 and 22 mRNA) was found in association with all transmembrane collagen, both mature and highly regularly fibrillar (mRNA of collagens 3, 11 and 24) and network-forming (mRNA of collagens 8 and 10) (Figure 1E). At the end of the healing period, 28 days post myocardial infarction, the number of α-SMA-positive myofibroblasts decreased, as expected. In the mature scar, we observed predominately collagen 11 mRNA, FACIT collagen 9 mRNA and network-forming collagen 10 mRNA gene expressions and fewer other collagen subtypes compared with the previous time points of the healing period (Figure 1E).

### 2.2. Immune Cells Influence Collagen Subtype Expression and Biomechanics after MI

**Figure 2:**
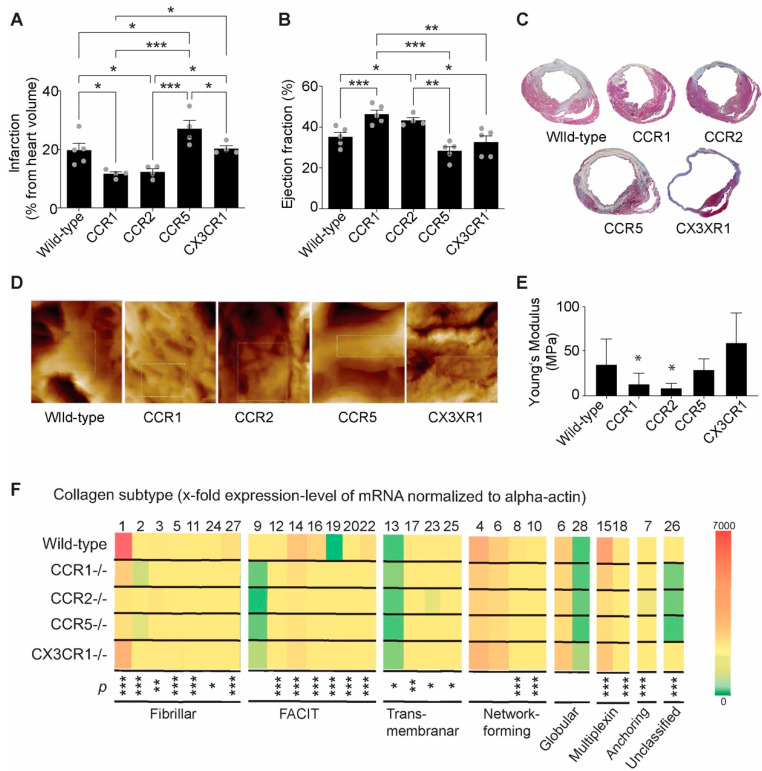
Assessment of infarction size and functional parameters of the heart after myocardial infarction. (**A**) Infarction size measured after Gomori’s one-step trichrome staining in a serial section in wild-type and different knockout mice four weeks after myocardial infarction (* *p* < 0.05, *** *p* < 0.001, one-way ANOVA, one-way ANOVA followed by Tukey’s post hoc test comparing the groups, n = 4–5 mice). (**B**) Echocardiographic measurements of ejection fractions 4 weeks after myocardial infarction (* *p* < 0.05, ** *p* < 0.01, *** *p* < 0.001, one-way ANOVA followed by Tukey’s post hoc test comparing the groups, n = 4–5 mice). (**C**) Representative Gomori’s one-step trichrome stain images of cross-sectioned tissues from wild-type and knockout mice 4 weeks after myocardial infarction. (**D**) Representative images of scar structures in atomic force microscopy topological images of scar microstructures (50 µm/50 µm) and examples of areas (inserts) considered for analysis (pores were avoided). (**E**) Scar tissue stiffness of different knockout mice analyzed by liquid atomic-force microscopy (* *p* < 0.05 vs. wild type, one-way ANOVA followed by Tukey’s post hoc test, n = 4–5 mice). (**F**) mRNA expression levels of different collagen subtypes 4 weeks after myocardial infarction (normalized to α-actin) in the various mutant mice (* *p* < 0.05, ** *p* < 0.01, *** *p* < 0.001, one-way ANOVA followed by Tukey’s post hoc test for each collagen subtype, n = 3 mice per group).

Previously, we showed the influence of neutrophils on extracellular matrix production and healing after myocardial infarction [6]. Herein, we investigated the contribution of different immune cells on scar formation via the expression of collagen subtypes. To this end, we employed various mouse models with different inflammatory patterns. We performed myocardial infarction in CCR1^−/−^ (reduced neutrophils recruitment [25]) mice, CCR2^−/−^ (reduced inflammatory monocyte recruitment [28,29]) mice, CCR5^−/−^ (reduced T-cell recruitment [31]) mice and CX3CR1^−/−^ mice (reduced reparatory monocyte recruitment [32]) mice. As previously established, there were significant differences in infarction size between the groups (Figure 2A). CCR1^−/−^ (11.7 ± 1.4%) and CCR2^−/−^ (12.3 ± 2.1%) showed a significant reduction in infarction size compared to the C57BL/6 wild-type control, whereas CCR5^−/−^ (20.2 ± 5.7%) did not differ, and CX3CR1^−/−^ (27.1 ± 2.0%) exhibited a significant increase in infarction size compared to the wild type (19.7 ± 5.1%). Similarly, the ejection fraction showed a preserved cardiac function in CCR1^−/−^ (46.3 ± 4.6%) and CCR2^−/−^ (43.3 ± 2.7%) mice but not in CCR5^−/−^ (32.7 ± 6.6%) and CX3CR1^−/−^ (28.4 ± 4.5%) mice after myocardial infarction induction compared to wild-type mice (35.2 ± 4.7%, Figure 2B), as assessed by echocardiography. Except for CX3CR1^−/−^ mice, which presented with massive diastolic dilatation, we did not observe other differences in ventricular dimensions in other groups four weeks after myocardial infarction (Figure 2C). Despite this dilatation, we observed that the remote part of the CX3CR1^−/−^ hearts seem to develop a certain grade of hypertropia compared with the wild type, probably due to the adaptation to increase dilatation of the infarcted area, mitigating the possible difference between the two groups when included in the calculations (the infarction volume was calculated and presented as percentage of entire heart volume). Similarly, we noted a hypercontraction of the myocardium in the remote regions of the CX3CR1 hearts, which likely prevented the significant decrease in ejection fraction compared with the wild-type mice. Further morphological and biomechanical modifications were assessed 4 weeks after myocardial infarction by atomic force microscopy under mimicking aqueous conditions (Figure 2D,E). At high resolution, the surface morphology of each tissue knockout mouse displayed specific morphological signatures compared to those of the control group. For example, CCR1^−/−^ and CCR2^−/−^ mice seemed to develop similar morphological maps of heterogeneous interwoven microstructures of thin and robust fibers (Figure 2D). However, the morphological map of CCR5^−/−^ mice displayed robust fibers, whereas CXCR3^−/−^ mice exhibited fibers with a heterogeneous morphology. Based on Young’s modulus estimations as a measure of regional tissue stiffness, we found that both CCR1^−/−^ and CCR2^−/−^ mice tissues were compliant compared to the control and other study groups (Figure 2E). These findings suggest that hearts with small infarction size and such heterogeneous microstructures contribute to the development of compliant tissue. On the contrary, when the matrix was structurally organized, it tended to limit the expansion of the fibrils, thereby interconnecting them with adjacent muscle fibers. As such, the regional tissue might appear to be stiff.

We further investigated the differential expression of collagen subtypes in the different knockout mice 28 days post infarction. To avoid differences in terms of the number of myofibroblasts, all values were normalized to alpha-actin expression. All collagen subtypes were found to be expressed in varying proportions in the final scar after myocardial infarction. However, significant differences in collagen subtype content between the experimental groups were observed (Figure 2F). Wild-type mice exhibited a significantly higher expression of collagen 1, 3, 12, 14, 15, 16, 22 and 27 than all other groups. Scar tissue from CCR1^−/−^ presented with high mRNA expression of collagen 11, moderate expression of collagen 19 and low expression of collagens 13 and 26 compared with other groups. CCR2^−/−^ scar tissue showed high levels of collagen 13, whereas CX3CR1^−/−^ scar tissue expressed high levels of collagen 19 and collagen 26 compared with other mice (Figure 2F).

### 2.3. Differential Role of Immune Cells in Proliferation/Apoptosis of Myofibroblasts

**Figure 3:**
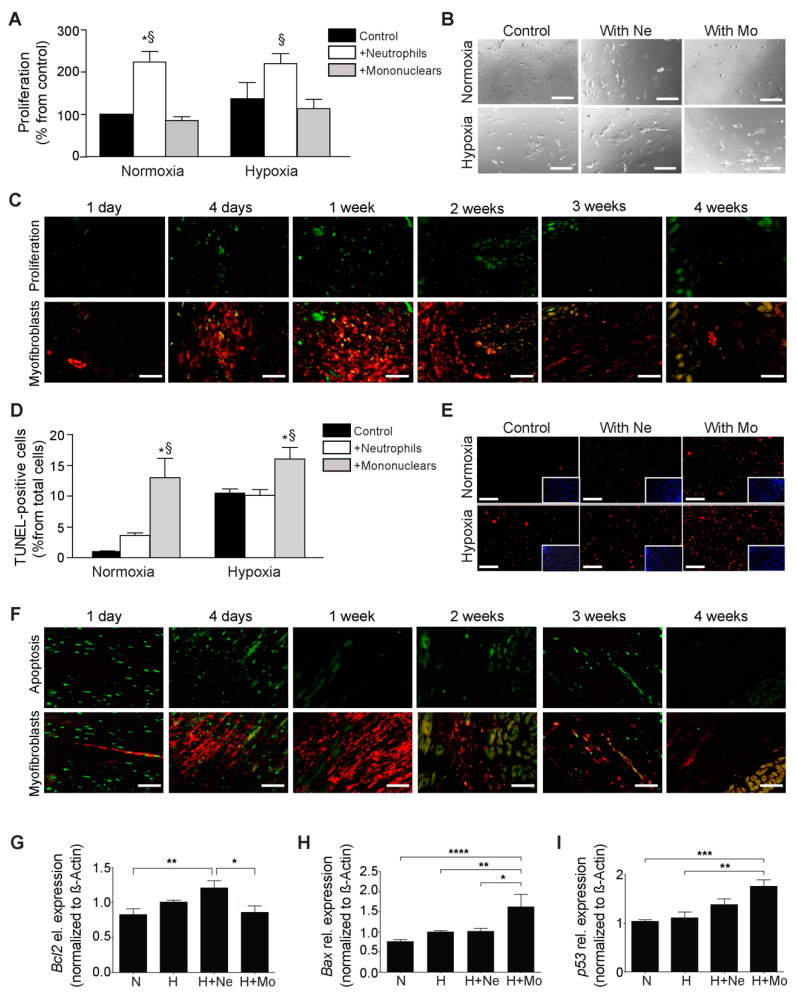
The influence of immune cells on apoptosis and proliferation of myofibroblasts. (**A**) Proliferation of myofibroblasts under normoxic/hypoxic conditions in the presence of neutrophils (Ne) and mononuclear cells (Mo) (* *p* < 0.05 vs. control, ^§^ *p* < 0.05 vs. mononuclear cells, one-way ANOVA followed by Tukey’s post hoc test comparing the groups, n = 5–6; the experimental data for one run are presented; the experiment was repeated four times). (**B**) Representative images of proliferating myofibroblasts in the presence of neutrophils and mononuclear cells (phase light; scale bar, 100 µm). (**C**) Representative images of proliferation (Ki67 in bright green, **upper panel**) of myofibroblasts (**lower panel**, Ki67 in bright green; alpha-actin in red; scale bar, 50 µm). (**D**) Apoptosis of myofibroblasts under normoxic/hypoxic conditions in the presence of neutrophils and mononuclear cells (* *p* < 0.05 vs. control, ^§^ *p* < 0.05 vs. neutrophils, one-way ANOVA followed by Tukey’s post hoc test comparing the groups, n = 7; the experimental data for one run are presented, the experiment was repeated three times). (**E**) Representative images of apoptotic myofibroblasts in the presence of neutrophils and mononuclear cells (apoptotic cells in red; scale bar, 100 µm; DAPI blue counterstaining in inserts showing the similar distribution of the cells in the cultures; a correction of the brightness/contrast was applied equally to all images in the power point to improve visibility for the reader). (**F**) Representative images of apoptotic cells (TUNEL in bright green, **upper panel**) of myofibroblasts (**lower panel**, TUNEL in bright green; alpha-actin in red; scale bar, 50 µm). (**G**) mRNA expression of *Bcl2* as a survival and antiapoptotic marker in myofibroblasts (* *p* < 0.05, ** *p* < 0.01, one-way ANOVA followed by Tukey’s post hoc test comparing the groups, n = 9; three samples from three runs in triplicate) under normoxia (N), hypoxia (H) in coculture with neutrophils (Ne) or mononuclear cells (Mo). (**H**) mRNA expression of *Bax* as ab apoptotic marker in myofibroblasts (* *p* < 0.05, ** *p* < 0.01, **** *p* < 0.0001, one-way ANOVA followed by Tukey’s post hoc test comparing the groups, n = 9; three samples from three runs in triplicate). (**I**) mRNA expression of *p53* as an apoptotic marker in myofibroblasts (** *p* < 0.01, *** *p* < 0.001, one-way ANOVA followed by Tukey’s post hoc test comparing the groups, n = 9).

The proliferation and apoptosis of myofibroblasts significantly impact scar formation after myocardial infarction. To dissect how immune cells might influence the proliferation and apoptosis of myofibroblasts after myocardial infarction, we incubated cardiac myofibroblasts with either neutrophils or mononuclear cells and performed proliferation and apoptosis staining. The coincubation with neutrophils significantly increased the proliferation of myofibroblasts (by almost double) compared to the control and mononuclear cell coincubation under normoxic and hypoxic conditions (Figure 3A,B). By staining myofibroblasts and proliferation in vivo during healing after myocardial infarction (Figure 3C), we observed the highest proliferation rate of myofibroblasts in initial phases four and seven days after myocardial infarction, corresponding with the recruitment of neutrophils.

Coincubation with mononuclear cells significantly increased the apoptosis of myofibroblasts compared to the control and neutrophil coincubation under normoxic and hypoxic conditions (Figure 3D,E). Despite the increases in the apoptosis rate due to hypoxia, incubation with mononuclear cells further significantly increased the apoptosis rate in myofibroblasts. The identification of apoptotic myofibroblasts in in vivo sections at multiple time points post infarction revealed that the apoptotic myofibroblasts can be found at later time points, i.e., at 14 and 21 days post infarction (Figure 3F), corresponding with the most abundant presence of mononuclear cells/macrophages, which can explain the deactivation and disappearance of myofibroblasts in the mature scar. The apoptotic cells observed one and four days post infarction are not stained for myofibroblasts markers, as they were mostly cardiomyocytes (Figure 3F).

These results were confirmed by investigating the mRNA expression of typical survival, antiapoptotic gene *Bcl2* and apoptotic genes *Bax* and *p53* in cocultured myofibroblasts. Similar to previous experiments, neutrophils significantly increased the expression of the *Bcl2* mRNA level in myofibroblasts compared with control and mononuclear coculture (Figure 3G). The coculture with mononuclear cells notably increased the expression of proapoptotic genes such as *Bax* (Figure 3H) and *p53* (Figure 3I) in myofibroblasts compared to the control and neutrophil coculture.

### 2.4. Differential Role of Immune Cells in the Biomechanical Properties of Myofibroblasts by Inducing Differential Extracellular Matrix Proteins and Collagen Subtype Synthesis

We further investigated the influence of neutrophils and mononuclear cells on the differential collagen synthesis of myofibroblasts in vitro.

**Figure 4:**
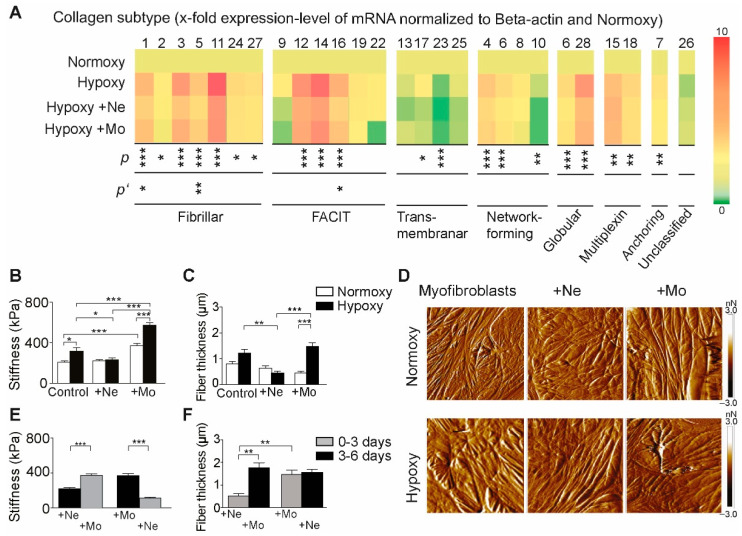
Assessment of mRNA expression of extracellular matrix proteins and biomechanics in mice four weeks after myocardial infarction. (**A**) qRT-PCR was performed for a semi-quantitative assessment of collagen subtypes in the absence/presence of different immune cells. Isolated myofibroblast were cultured under normoxic and hypoxic conditions and in coculture with neutrophils (+Ne) or mononuclear cells (+Mo). A heat map representing the level of mRNA expression was created in PowerPoint; significant values after ANOVA analysis of each protein are shown (* *p* < 0.05; ** *p* < 0.01; *** *p* < 0.001, one-way ANOVA followed by Tukey’s post hoc test, n = 3 mice). Significant differences between coincubation with mononuclear cells and neutrophils are shown separately (* *p*’ < 0.05; ** *p*’ < 0.01, one-way ANOVA followed by Tukey’s post hoc test, n = 3). (**B**) Myofibroblast stiffness in the absence and presence of neutrophil and mononuclear fractions, respectively (* *p* < 0.05, *** *p* < 0.001, one-way ANOVA followed by Tukey’s post hoc test, n = 3 mice). (**C**) Fiber thickness in myofibroblasts in the absence and presence of neutrophil and mononuclear fractions, respectively (** *p* < 0.01, *** *p* < 0.001, one-way ANOVA followed by Tukey’s post hoc test, n = 3). (**D**) Representative AFM images of myofibroblasts coincubated with immune cells under normoxia and hypoxia. (**E**) Myofibroblast stiffness after successive incubation steps with neutrophil followed by mononuclear fractions and with mononuclear followed by neutrophil fractions (*** *p* < 0.001, one-way ANOVA followed by Tukey’s post hoc test, n = 3 mice). (**F**) Fiber thickness in myofibroblasts after successive incubation steps with neutrophil followed by mononuclear fractions and with mononuclear followed by neutrophil fractions (** *p* < 0.01, one-way ANOVA followed by Tukey’s post hoc test, n = 3 mice).

Isolated myofibroblasts were differentiated and cultivated under normoxic and hypoxic conditions with/without coincubation with immune cell subsets. Hypoxic conditions induced dramatic changes in mRNA expression of 20 of 29 collagen subtypes (Figure 4A). Coincubation with inflammatory cells also changed the mRNA expression of collagen 1, 2, 3, 4, 5, 6, 7, 10, 11, 12, 14, 16, 17, 23 and 28 in myofibroblasts. Whereas coincubation with neutrophils reduced the level of collagens 1, 3, 5, 16 and 23, mononuclear coincubation reduced the expression of collagens 5 and 16 (Figure 4A). These results demonstrate that myofibroblasts are specialized cells with high versatility/adaptability not only for the synthesis/production collagen but also to modulate or periodically regulate collagen composition as a response to regional modifications induced by unknown mechanisms.

Because any changes in myofibroblasts significantly affect the biomechanics of the scar and subsequent heart function, we further investigated the local mechanical properties of myofibroblasts (Young’s modulus) at the nanoscale, as well as the contractile fiber thickness using liquid PFT-QNM-AFM [33]. Increased levels of, e.g., high tensile strength and rigidity of fibrillar collagen matrix, regulate not only the dynamic interplay between cells but with the matrix and among adjacent cells [34], thereby modulating the mechanical function and morphology of cells. Therefore, we first sought to define nanoscale modifications of cell stiffness and the thickness of their cytoskeleton filaments under hypoxia vs. normoxia. Notably, the mononuclear cells induced increased stiffness in myofibroblasts, even under normoxic conditions, with a further twofold increase in modulus values under hypoxic conditions (Figure 4B). As expected, the control became stiffer when culturing in hypoxia than in normoxia (Figure 4B). This finding suggests the accumulation of increased levels of high-tensile-strength fibrillar collagen, which seems to be associated with increased fibril thickness (Figure 4C). One expectation of increased fibrils dimensions is the heterogeneous cytoskeleton of prominent aligned filaments and the production of high internal stress as recorded in peak force-tapping AFM mode (Figure 4D). The moderate stiffness levels of cells in the case of +Ne coincubation might indicate the adaptation of myofibroblasts to the soft collagen matrix, as mRNA fibrillar collagen levels were reduced drastically (Figure 4A). Myofibroblasts tune their internal stiffness towards small values to match that of the collagen matrix, exhibiting small fibrils (Figure 4C). As cell-modulation implies a dynamic and reversible process, we assessed whether the changes in biomechanics induced by neutrophils and mononuclear cells are permanent or can be changed. Thus, the myofibroblasts cocultured with neutrophils were washed and further cocultured with mononuclear cells, whereas myofibroblasts cocultured with mononuclear cells were cocultured with neutrophils for another 72 h. We observed that the changes in mechanical properties induced by different immune cells are completely reversible, confirming their ability to modulate the plasticity of myofibroblasts (Figure 4E)**.** However, the changes induced by mononuclear cells are more robust and probably need more time to reverse their structural effects, as the increased fiber thickness and their alignment induced by mononuclear cells could not be reversed by neutrophils during the incubation time (Figure 4F).

### 2.5. Signaling Pathways Involved in Collagen Expression

**Figure 5:**
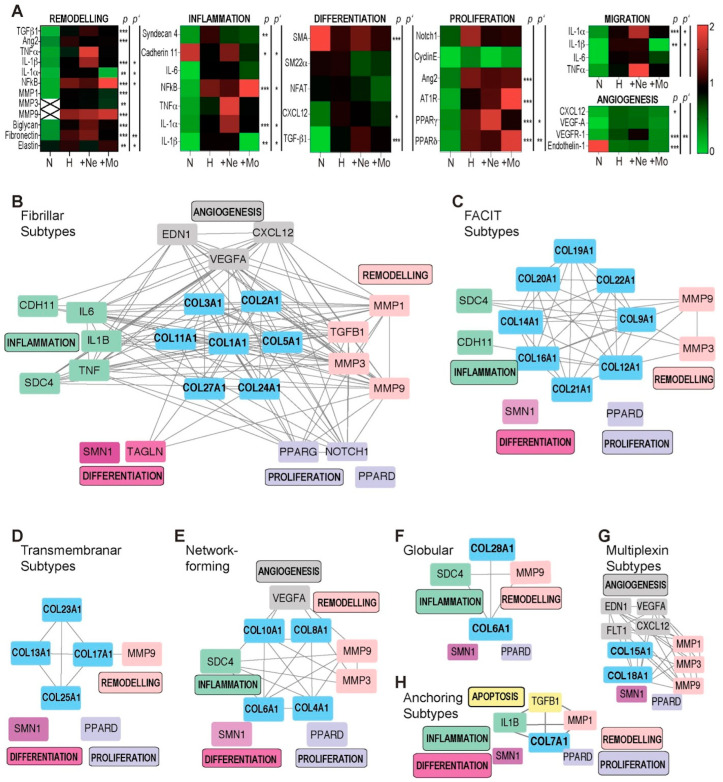
The modulation of gene expression by immune cells. (**A**) qRT-PCR was performed for a semi-quantitative assessment of collagen subtypes in the absence/presence of different immune cells. Isolated myofibroblasts were cultured under normoxic (N) and hypoxic conditions (H) and in coculture with neutrophils (+Ne) or mononuclear cells (+Mo). A heat map representing the level of mRNA expression was created in PowerPoint; significant values after ANOVA analysis of each protein are shown (* *p* < 0.05; ** *p* < 0.005; *** *p* < 0.0005, one-way ANOVA followed by Tukey’s post hoc test, n = 3; the experimental data for one run are presented; the experiment was repeated five times). Significantly differences between coincubation with mononuclear cells and neutrophils are shown separately (* *p*’ < 0.05; ** *p*’ <0.01, one-way ANOVA followed by Tukey’s post hoc test, n = 3; the experimental data for one run are presented; the experiment was repeated five times). (**B**–**H**) Interactions between signaling pathways and collagen subtypes identified in the gene–protein interaction analysis.

To identify the mechanisms and the signaling pathways involved in the differential modulation of the synthesis of collagen subtypes in myofibroblasts, the mRNA expression of different clusters of genes were analyzed (remodeling-related genes, differentiation-related genes, migration-related genes, proliferation-related genes, apoptosis-related genes, inflammation-related genes and angiogenesis-related genes). Most of the genes seem to be affected differently by immune cells (Table 1, Figure 5A). Whereas neutrophils activated the IL-1 pathway and PPARγ, mononuclear cells upregulated the NF-kB pathway, angiotensin II and PPARδ. A systematic and comprehensive review of different collagen clusters in relation to different genes sets revealed that genes related to remodeling interfered, as expected, with all types of collagen clusters (Figure 5B–H). Inflammation-related genes were mostly modulated by the fibrillar collagen subtypes (Figure 5B) and less influenced by FACIT (Figure 5C), network-forming (Figure 5E) and globular subtypes (Figure 5F). Proliferation and differentiation genes seemed to be associated only with fibrillar collagen subtypes (Figure 5B), whereas angiogenesis-related genes are associated with fibrillar (Figure 5B), network-forming (Figure 5D) and multiplexin subtypes (Figure 5G).

## 3. Discussion

In the present study, we demonstrated that the myofibroblasts involved in myocardial infarction healing are mainly of local origin and can rapidly adapt their function in response to environmental changes. Thus, immune cells can modulate their function during healing after myocardial infarction, controlling collagen synthesis and deposition. Whereas we intend for findings to deepen the understanding of the mechanism of healing after myocardial infarction and scar formation, the process of collagen synthesis is highly complex, and further intensive investigation is needed to put together all the missing puzzle pieces in this still incipient knowledge process. 

Myofibroblasts are the main source of extracellular matrix protein and responsible for the replacement of the destroyed heart tissue with a scar after myocardial infarction [35,36]. Although fibrosis is interpreted as a negative process and there is a general trend in establishing therapeutical strategies to reduce fibrosis (concerning interstitial and perivascular fibrosis) [36,37,38], in the case of myocardial infarction, the rapid formation of scar tissue is essential to the maintenance of the mechanical integrity and function of the heart [39,40]. The phenotypic alterations of infarct myofibroblasts during scar formation may reflect changes in the surrounding matrix [3,41], which end when the density of myofibroblasts decreases in the mature scar as a result of activation of apoptotic pathways [42,43]. However, whether these phenotypic are caused by myofibroblasts of varying origin [44,45] that are present in the healing area at different time points or by changes in the same cell remains unclear. In addition to resident fibroblasts or local endothelial cells [35,46], bone-marrow-derived progenitor cells have also been described as a relevant source of myofibroblasts at the site of cardiac injury [45,46]. Using irradiated mice that received green fluorescent protein (GFP)-expressing bone marrow cells before myocardial infarction, we demonstrated that locally derived myofibroblasts can exert a significant influence on extracellular matrix production during healing after myocardial infarction. Recently, by investigating the single-cell mRNA spatial map of human cells involved in myocardial infarction [47], distinct populations of fibroblasts were identified; however, these populations seem to differ only in terms of their differentiation states (myofibroblast progenitors or terminally differentiated myofibroblasts) [47], suggesting that myofibroblasts can modulate and change their function and synthesis during the healing process, reflecting changes in the surrounding matrix.

Myofibroblasts play an important role in wound contraction and are the main source of collagen in a healing infarct [35]. As we have shown, collagen content in the healing infarct progressively increases with time and the activation of myofibroblasts. The formation of a mature scar comprising dense crosslinked collagen enhances the tensile strength of the infarct, in addition to increasing passive stiffness and contributing to diastolic dysfunction [11,12,14]. Recent studies have described “good” and “bad” collagens [48] related to fibrotic processes; each collagen transmits unique signals to surrounding cells, controlling their migration, proliferation and apoptosis, impacting organ function and its potential to progress or reverse [48]. Collagen is a generic term including 28 subtypes: fibril-forming (1, 2, 3, 5 and 11) which provide the 3D framework for all organs and tissues [13]; fibril-associated (9, 12, 14, 16 and 19), which modulate the biomechanical properties of the tissues and offer a structurization of the extracellular matrix [49]; network-forming (4, 8 and 10), which provide support and anchoring for cells, as well as a filter or barrier [50]; transmembrane (13 and 18), which are both extracellular matrix components and receptors for cell adhesion [51]; and or other tissues [30], the role of which is relatively unknown (Supplementary Table S1). Unlike collagen 3, collagen 1 is broadly studied as a marker of scar stability, as a lack of collagen 1 is associated with premature cardiac rupture [52]. Whereas collagens 1 and 3 have been the traditional focus of normal and infarcted extracellular matrices, other studies have shown that non-fibrillar collagens, such as collagens 4 and 6, might also play an important role in the differentiation and organization of the matriceal network of myofibroblasts [53,54]. Furthermore, modifying the presence of different collagen subtypes in knockout mice or using antibodies has significant effects on heart development and pathology, such as collagens 5, 11 [55], 13 [56,57], 14 [58], 15 [59] and 8 [60]. Other collagen subtypes are present in heart tissue, such as collagens 16 [61], 17 [62], 21 [63] and 27 [64]. However, their role in heart disease is unknown. In this study, we showed that all collagen subtypes are synthesized during healing after a myocardial infarction. Initially, transmembrane collagens and some FACIT collagens (collagens 9 and 19) are rapidly synthesized after myocardial infarction. This implies the necessity of rapid intercellular connection and signaling intermediated by transmembrane collagens, as well as the maintenance of the structure and biomechanics of the degrading area after myocardial infarction by FACIT collagens. Later, all collagen subtypes are expressed, peaking around 2 weeks after myocardial infarction but decreasing significantly at the end of the healing process. Just a few collagens remained highly expressed after 28 days, including collagens 1, 11 (fibrillar), 9 (FACIT) and 10 (network-forming). This is an important finding, suggesting that collagen subtypes are required for the synthesis of stable scar tissue, although they do not play a structural role in the final process. 

Furthermore, we demonstrated in our previous studies that the composition of the scar can be significantly modified by immune cells [6,25,26]. Using different knockout models, we showed that the collagen content can vary, despite the increased preservation of heart function or decreased infarction size. CCR2^−/−^ mice, which exhibit reduced inflammatory monocyte recruitment [28,29], showed preserved heart function and reduced infarction size after the induction of myocardial infarction. However, CCR1^−/−^ and CXCR4^−/−^, which exhibit reduced neutrophil recruitment [25,26], demonstrated an increased content of collagen in the infarcted areas, despite preserved heart function and decreased infarction size, suggesting a direct effect of immune cells on the collagen synthesis of myofibroblasts. In the current study, we investigated the collagen subtypes in different knockout models with a varying pattern of inflammatory response. As previously established, we observed a preserved ejection fraction and decreased infarction size in the mice presenting with reduced neutrophil infiltration (CCR1^−/−^) and reduced inflammatory monocyte infiltration (CCR2^−/−^), as well as depreciated ejection fraction and increased infarction size in mice with reduced reparatory monocyte infiltration (CX3CR1^−/−^) or T-regulatory cell infiltration (CCR5^−/−^) compared with wild-type mice undergoing a myocardial infarction procedure. Our results are also consistent with reduced stiffness as a requirement for a improved functional outcome after myocardial infarction (CCR1^−/−^ and CCR2^−/−^). However, the investigation of different collagen subtypes in the mature scar in these mice resulted in inconclusive results. Despite the differences between the groups, none of the collagen subtypes was directly associated with a specific group of mice. Thus, we can speculate that no singular collagen subtype can significantly influence the resulting mature scar, although the interconnection between different types of collagen subtypes should be considered and further investigated. Intensive research will be necessary to identify the role of each collagen subtype and the type of interconnection with other collagen subtypes to create such a simulation and to fully understand how the extracellular matrix is formed after myocardial infarction.

Moreover, when dissecting in vitro, the direct effects of immune cells on myofibroblasts under conditions mimicking the post-infarction environment, we reported significantly different results compared to those obtained in in vivo results, demonstrating that isolation of myofibroblasts and the investigation outside of the tissular environment could represent a major problem, suggesting that a careful interpretation of in vitro data and their translational relevance is required. 

It seems that hypoxic conditions activate various molecular pathways in myofibroblasts, including extracellular matrix protein production, inflammatory cytokine expression, proliferation, remodeling, etc. Notably, the neutrophils and mononuclear cells demonstrated differential modulatory capacity and were able to specifically and significantly manipulate the function and behavior of the myofibroblasts. 

As previously demonstrated, neutrophils and mononuclear cells are both required to interfere and actively determine the faith of myofibroblasts, thereby influencing scar formation after myocardial infarction [6]. Thus, directly after induction of myocardial infarction, fibroblasts start to differentiate and proliferate under the modulation of neutrophils, whereas collagen synthesis is impaired, probably mediated via PPAR-γ [65]. In vivo, the proliferative signals in myofibroblasts are present until one week after myocardial infarction and disappear completely thereafter, together with neutrophils. In addition, we demonstrated the role of mononuclear cells in switching to collagen synthesis in myofibroblasts, which begins the second week after induction of myocardial infarction to produce large amounts of collagen. In later phases, they are responsible for stopping proliferation and inducing apoptosis in myofibroblasts. Although there studies have shown that mononuclear cells are associated with the proliferation and matrix synthesis in myofibroblasts [32], we demonstrated here that mononuclear cells induce the apoptosis of myofibroblasts, thus actively influencing the maturation of the scar. Investigation of classical genetic markers of apoptosis inhibition or apoptosis, such as Bcl2, Bax or p53, support our data. Moreover, we observed an upregulation of PPARδ in myofibroblasts after coincubation with mononuclear cells, which is known to inhibit cardiac fibroblast proliferation, as well as fibroblast differentiation towards myofibroblasts [66]. In vivo staining showed a colocalization of apoptotic signals with myofibroblasts at later time points, i.e., three weeks after myocardial infarction, supporting the in vitro data. Thus, our data demonstrate that mononuclear cells induce apoptosis in myofibroblasts, in addition to switching temporarily to collagen synthesis under both normoxic and hypoxic conditions, highlighting, for the first time, that mononuclear cells could be responsible for the apoptosis of myofibroblasts in mature scars. 

The neutrophils sustain the upregulation of inflammatory pathways in myofibroblasts, such as the upregulation of interleukin 1 (both alpha and beta), which are known to be involved in dilative remodeling and post-infarction heart failure [67]. NF-kB and angiotensin II pathways are not influenced by neutrophils but by mononuclear cells. Under mononuclear coincubation, the myofibroblasts increased upregulation of NF-kappaB and angiotensin II pathways, downregulating PPAR-γ and increasing collagen synthesis [65,68,69], in accordance with our findings. Collagen subtypes 1, 5 and 16 were upregulated in the presence of mononuclear cells under hypoxic conditions. We observed that in addition to collagen I, all other subtypes were expressed and induced during scar formation; however, their role is yet to be understood and likely cannot be elucidated using existent methods, owing to the complexity of interaction between all the extracellular matrix components. In this context, the results obtained by analyzing the collagen subtypes after incubation of isolated myofibroblasts with different immune cells are inconclusive and difficult to understand, considering the current state of the art. 

By analyzing cell microelasticity, which is considered to be one of the primary mechanical parameters describing the regulation of cell functions, we observed significant decrease in the stiffness of myofibroblasts coincubated with neutrophils. This effect was promptly reversible when myofibroblasts were subsequently coincubated with mononuclear cells. Owing to the lack of knowledge about the correlation of cell activity with biomechanics, it is difficult to speculate on the significance of these data. However, because cell elasticity is modulated by several factors, such as the reorganization of cytoskeleton elements at the micron level, including actin filaments, microtubules and intermediate filaments, we can understand that the effect of the modulation of immune cells on myofibroblasts reaches beyond their function and signaling, also including structural and molecular changes in the cytoplasm and organelles of the myofibroblasts. The extent to which these changes can be modulated is still unknown and remains to be investigated.

Surprisingly, we observed a correlation between the different collagen subtypes forms and clusters of genes. As expected, the remodeling-associated genes are associated with all collagen subtypes. However, fibrillar collagens influence most clusters of genes, including inflammatory, angiogenesis, differentiation and proliferation genes, whereas other collagen subtypes specifical influence just one or two clusters of genes. This finding is also important, demonstrating that the formation of specific collagen subtypes during healing can modulate transitory specific functions, which cease when scarring is complete. However, further complex investigations are necessary to understand the specific role of each player in this process.

**Limitations**. Our study is subject to several limitations, which we address below. First, we are aware that, owing to the complexity of the collagen structure, this study is represents only an overview of the potential directions to be considered when studying the extracellular matrix during healing after myocardial infarction. Separate investigations for each collagen subtype, as well as other extracellular matrix proteins, should be performed to comprehend all the biological processes involved, which was not the aim of this study. Furthermore, owing to the lack of specific antibodies for each collagen subtype, we were not able to validate our findings with respect to protein levels. mRNA expression does not reflect protein production, as various factors, such as degradation, etc., might influence transcription. However, we believe that our data can provide a broad orientation and pave the way for more in-depth studies. Second, owing to the multitude of players involved, bioinformatic methods of analysis should be implemented to integrate all the information obtained from all individual studies. Furthermore, an appropriate model that can better mimic in vivo hypoxia conditions should be developed, as myofibroblasts are extremely sensitive to environmental changes. Third, owing to the preliminary nature of this study, we adhered to the novel 3R regulations and attempted to use the minimal number of mice that would allow us to present an overview and create a path for future investigations, which should repeat any of these experiments in a more focused and detailed manner. Fourth, although we expect to see effects of the reduced number of specific cells in our knockout mice on collagen subtype expression, we cannot exclude confounding factors affecting collagen expression as a result of unknown effects of the knockout. 

**Conclusion.** In conclusion, the research presented herein is pilot study offering an overview of the complexity of collagen synthesis during the healing process after myocardial infarction, in which myofibroblasts play a central role. After the ischemic event, the hypoxic conditions activate the fibroblasts, sustaining their differentiation towards myofibroblasts. Neutrophils additionally increase differentiation and inhibit apoptosis of myofibroblasts, reducing their collagen synthesis and sustaining a rapid and efficient synthesis of a provisional fibrin-rich matrix. Later, monocytes modulate the myofibroblasts, switching to collagen synthesis and a collagen-based matrix. Collagen subtypes seem to play varied roles in scar formation during healing processes. Collagen synthesis is a transitory process; reparatory monocytes induce apoptosis signals in myofibroblasts in the late phase of healing after myocardial infarction, thus stabilizing and maturating the scar. Because all these processes involve multiple players and factors, the role of each component of the extracellular matrix should be reconsidered and reanalyzed in the complex context of matrix–cell interactions.

## 4. Material and Methods

All animal experiments were performed in accordance with European legislation and approved by local German authorities (LANUV—Landesamt für Natur, Umwelt und Verbraucherschutz Nordrhein-Westfalen, approval number: AZ: 8.87-50.10.35.09.088; approval date: 1 June 2009). All mice were housed under standardized conditions in the Animal Facility of the University Hospital Aachen (Aachen, Germany).

### 4.1. Animal Model of Myocardial Infarction (MI) Induction

Eight-week-old male C57BL/6 wild-type mice, as well as CCR1^−/−^ mice (Charles River Germany, Sulzfeld, Germany) with known reduced neutrophil infiltration [25], CCR2^−/−^ mice (kindly provided by Prof. M.W. Merx) with known reduced inflammatory monocytes infiltration [29], CCR5^−/−^ mice (Charles River Germany, Sulzfeld, Germany) with known reduced reparatory monocyte and T-regulatory cell infiltration [31,32] and CX3CR1^−/−^ mice (Charles River Germany, Sulzfeld, Germany) with known reduced reparatory monocyte infiltration [32] underwent chronic myocardial infarction (n = 5–6 per group) as described previously [70]. Briefly, mice were intubated under general anesthesia conditions (100 mg/kg ketamine, 10 mg/kg xylazine, i.p.) and ventilated with oxygen using a mouse respirator (Harvard Apparatus, Germany). After exposing the hearts by left thoracotomy, myocardial infarction was induced by occlusion of the left anterior descending artery (LAD) with a 0/7 silk suture. The ribs, muscle layer and skin incision were closed, and morphine (buprenorphine 0.1 mg/kg) was administered until full recovery.

#### 4.1.1. Echocardiography

Four weeks after myocardial infarction, the left ventricular ejection fraction was determined by acquiring two-dimensional (long and short axis) and M-mode (long and short axis) echocardiographic imaging using a small-animal ultrasound imager (Vevo 770, FUJIFILM VisualSonics, Toronto, ON, Canada). Finally, the hearts were excised and prepared for further analysis, as previously described [71].

#### 4.1.2. Bone Marrow Reconstruction

To elucidate the origin of fibroblasts, we conducted bone marrow transplantation experiments using bone marrow from GFP-positive mice. Six-week-old wild-type (C57BL/6) male mice from Charles River Laboratories (Cologne, Germany) (n = 3 per group) were lethally irradiated with 2.0 Gray divided into two doses as described previously [25] and reconstituted with 1 × 10^6^ green fluorescent protein (GFP)-positive bone-marrow cells isolated from femurs and tibias of GFP transgenic mice (kindly provided by Prof. M. Zenke). Six weeks after reconstitution, the mice underwent myocardial infarction as previously described [70]. Their hearts were excised 4 weeks after myocardial infarction and prepared for further analysis.

#### 4.1.3. Histology and Immunohistochemistry

Serial sections (10 sections per mouse, 400 µm apart, up to the mitral valve) were stained with Gomori’s one-step trichrome stain. The blue-stained infarcted area was determined for all sections using Diskus software (Hilgers, Königswinter, Germany), and the volume of the infarcted heart was calculated and expressed as a percentage of total left ventricular volume [70]. Furthermore, collagen fibers were stained with Picro-Sirius red, imaged and acquired in polarized light, enabling observation of thickness of the collagen fibers; the young, thin fibers appear as green under polarized light, whereas during maturation and thickening, the color changes to orange–red [72]. Myofibroblasts were stained using an anti-smooth muscle actin antibody (SMA, DAKO, Hamburg, Germany) followed by an alkaline phosphatase kit (Vector Laboratories, Germany). The origin of the myofibroblasts was assessed for serial sections after double staining using GFP antibody (Abcam, Cambridge, UK) followed by FITC-conjugated secondary antibody and smooth muscle actin (SMA, DAKO, Hamburg, Germany) and an alkaline phosphatase kit (Vector Laboratories, Eching, Germany). DAPI was used as a nuclear counterstain. GFP^+^, SMA^+^ and GFP/SMA double-positive cells were counted and expressed as cells/power fields. TUNEL staining was performed according to the manufacturer’s protocol (In Situ Cell Death Detection Kit, Sigma-Aldrich, Darmstadt, Germany) in serial sections, followed by double staining with SMA. Proliferation staining was performed using a Ki-67 antibody (Thermo Fisher Scientific, Germany, RM-9106) followed by anti-rabbit FITC-conjugated antibody (Abcam, Cambridge, UK) and double staining with SMA. To differentiate the smooth muscle cells from myofibroblasts, all alpha actin-positive cells inside of the structure of the vessel were excluded. The remaining cells were counted based on DAPI counterstaining of the section.

#### 4.1.4. Myofibroblast Isolation and Differentiation

Adult murine hearts (left ventricle) were mechanically triturated in Dulbecco’s modified Eagle medium (DMEM) buffer and Liberase (Liberase Blendzyme 1, Roche) and incubated under shaking (400 rpm, 37 °C, 25 min). The obtained cell suspension was filtered using a cell strainer (BD Falcon cell strainer 100 µm Nylon, REF 352360), centrifugated (400× *g*, 20 °C, 5 min) and cultured in high-glucose DMEM (4.6 g/L) with L-glutamine (PAA-The Cell Culture Company, Minneapolis, MN, USA), 10% FBS (fetal bovine serum dialyzed, PAN Biotech, Aidenbach, Germany) and 1% penicillin/streptomycin (PAA-The Cell Culture Company). After 4 h of incubation, the medium was removed, together with the still-floating cells, with only fibroblasts able to adhere and survive under these conditions. The adherent fibroblasts were allowed to reach confluence. The differentiation of fibroblasts into myofibroblasts was induced with 100 ng/mL TGF-β1 and hypoxia (5% CO_2_, 2% O_2_, 37 °C, Innova^@^ CO-48 Incubator) to mimic in vivo hypoxia conditions. Coculture was performed by adding neutrophils and mononuclear cells isolated from mouse blood using Lympholyte (1086 g/cm^3^, Cedarlane, Burlington, ON, Canada) as recommended by the manufacturer. After 24 h, the cells were washed with PBS and used for further analysis. Each experiment was run in triplicate for each sample. Representative results obtained in a single run are presented (excluding the runs resulting from defects in amplifications). For each in vitro experiment, the myofibroblasts were isolated from different mice (at least three mice per group). If myofibroblasts were pooled together, then the experiments were conducted in three to five runs (different days), as indicated in the figure legend; the data for a single, representative run are presented.

#### 4.1.5. mRNA Isolation and RT-PCR

mRNA was isolated from mouse infarcted areas and cultured myofibroblasts using an RNeasy Mini Kit (Qiagen, Hilden, Germany). mRNA expression of collagen subtypes and other genes was quantitatively determined using specific primers (Table 2) and murine ß-actin as a housekeeping gene. We used murine ß-actin as a housekeeping gene, although glyceraldehyde-3-phosphate dehydrogenase (GAPDH) has been used as a housekeeping gene in other studies investigating fibrosis [73,74], owing to the possible similarities between β- and α-actin. However, other studies show a variation of up to 40% of GAPDH mRNA expression under hypoxic conditions [72,75,76,77]. In the current study, because we compared the quality of the fibrotic tissues under hypoxic and normoxic conditions and not the quantity, we decided to use β-actin as housekeeping gene and to subsequently normalize values to α-actin when possible and indicated. Each experiment was run in triplicate for each sample. Each in vitro experiment was repeated on three different days by three different investigators. Representative results obtained in a single run by the same investigated are presented (excluding the runs resulting from defects in amplifications). In vivo experiments were conducted with n = 3 animals in triplicate.

#### 4.1.6. Isolation of Fibroblast Precursors from Blood and Myofibroblast Differentiation

Peripheral blood mononuclear cells were isolated from blood using Lympholyte (1086 g/cm^3^, Cedarlaine, Burlington, NC, USA) as recommended by the manufacturer. They were cultivated on Petri dishes for 24 h. Differentiation was induced with 100 ng/mL TGF-β1 under hypoxia (5% CO_2_, 2% O_2_, 37 °C, Innova^@^ CO-48 Incubator) to mimic in vivo hypoxia conditions. Then, cells were stained with anti-smooth muscle actin antibody (SMA, DAKO, Hamburg, Germany) followed by anti-mouse FITC (Abcam, Cambridge, UK). The stained and unstained cells were quantified by FACS and expressed as percentage positive cells among total cells. Each in vitro experiment was repeated three to five times as indicated. 

### 4.2. In Vitro Evaluation of the Influence of Monocytes/Neutrophils on the Apoptotic/Proliferation Process of Mouse Cardiac Fibroblasts Subjected to Hypoxic Conditions

Mouse cardiac myofibroblasts, neutrophils and mononuclear cells were isolated and cultured as described above. Before proceeding with hypoxia exposure, the myofibroblasts were seeded at a cell density of 6 × 10^4^ cells/1.9 cm^2^ and incubated for 24 h under normoxic cell culture conditions (37 °C and 5% CO_2_). Next, the media were replaced with hypoxic media with or without TGF-β1 to a final concentration of 15 µg/mL, and the cells were further incubated under hypoxia (5% CO_2_, 2% O_2_, 37 °C, Innova@ CO-48 Incubator) with a suspension of neutrophils and mononuclear cells for 48 h. In parallel, this setup was conducted under normoxic conditions as a control. At the end of the experiment, cells were fixed with 2% paraformaldehyde (P-6148, Sigma, Darmstadt, Germany) and subjected to another TUNEL assay (Rosche 12156792910, Penzberg, Germany) for apoptosis evaluation. The cell culture dishes were photographed using DISKUS (Hilgers, Germany) for the TUNEL assay. Positively stained cells were counted in six to eight different fields per cell culture dish and expressed as a percent of positive cells among total cells per field of view (20-fold magnification). Representative images are shown. The experiments were repeated concomitantly in different six-well Petri dishes. The results represent the average of each well for each condition.

Additionally, the proliferation of myofibroblasts under the same cell culture conditions as described above was evaluated using a CyQUANT cell proliferation assay kit (LifeTechnologies, REF C7026). Proliferation was quantified according to the CyQUANT cell proliferation assay kit assay using a Tecan reader. The results are presented as arbitrary fluorescent units for at least three replica experiments. Confluent wells were excluded from analysis. Representative results obtained on the same day are shown. Each in vitro experiments was repeated at least three times.

#### 4.2.1. Atomic Force Microscopy (AFM) Topography and Biomechanics Assay

The topography of cells and heart tissues was recorded by means of peak force tapping-quantitative nanomechanics-AFM (BioScope, Bruker, Santa Barbara, CA, USA). Prior to AFM measurements, cells were directly seeded on a muscovite mica surface and carefully washed with 2 × 12 mL PBS to remove potential cell debris floating in the path of the laser. AFM measurements were conducted in PBS. Topography images (3 images/sample) of living cells were obtained in a raster pattern in the peak force error channel to enhance the contrast of the fibrils at the nanoscale level. In brief, the peak force error channel generates a map of the peak force by sensing the force change between a V-shaped sharp stylus probe (nominal spring constant, 0.07 N/m; nominal length, 180 mm; nominal tip radius at apex, 12 nm; and resonant frequency, 22 kHz) and the specimen surface. A scan rate and pixel per line of 0.5 Hz and 512, respectively, were chosen to minimize the cell surface damage while keeping the contact between the tip and cell surface. Image quality (minimization of lateral forces exerted by the scanning tip, etc.) was achieved by further reducing the scan rate to 0.2 Hz. A low-pass Gaussian filter applied along the X and Y axes ensured increased detail at the mesoscale. Microscope slides of deparaffinized and unstained heart tissue sections were placed on the AFM stage and imaged using Bruker V-shaped etched silicon tips (nominal spring constant, 5 N/m; resonant frequency, 150 kHz; nominal tip radius, 12 nm). Three images per tissue section were recorded in PBS at a scan rate of 0.95 Hz, and the resolution was set at 256 pixels per line for a fixed scan size of 50 × 50 mm^2^. Given the inherent roughness of the heart tissue samples, surface tracking and its quality were optimized by reducing the scan rate to 0.5 kHz, with scanning performed along the length of myocardial cells [1].

Topology maps were recorded simultaneously with stiffness maps, thus enabling the association of any local morphological and biomechanical correlation. The peak force QNM method facilitates the acquisition and analysis of individual force–displacement curves recorded during cell imaging. The precontact point was previously determined to exclude the non-contact and artefact regions from the Young’s modulus calculation. The radius of curvature of the AFM tip was calculated by engaging the tip onto a polycrystalline titanium rough surface, whereas the deflection sensitivity of the cantilever was determined against a clean glass slide. Based on a single-ramp test of deflection error vs. displacement, the deflection sensitivity was calculated according to the linear regime, whereas the resonance frequency of the cantilever was determined based on the thermal tune method [78]. The resonant peak was fit with a Lorentzian function and provided a force constant of the cantilever of 0.174–0.266 N/m. Force–distance curves were generated 10–20 times at two or three different locations on each specimen. Individual force–distance curves were plotted and further analyzed by means of the Derjaguin–Muller–Toporov (DMT) model [78]. The results for each separated group were further aggregated and compared. Notably, stiffness and structure of heart tissue might be altered by sample preparation. For example, heart tissue might shrink and induce significant loss of protein phosphorylation due to the formalin fixed-paraffin embedding processes, leading to possible modifications at the nanoscale. However, we assumed that these effects vary across all group samples. As such, the relative comparison of results is relevant, and their statistics can be considered [1].

Owing to the complexity of the experiments and limited access to equipment, these experiments were conducted in a single run for n = 3 myofibroblasts cultures isolated from different mice.

#### 4.2.2. Reverse Incubation of Myofibroblasts

After 72 h of incubation with a blood cell type and AFM measurement of stiffness, the supernatant was removed and washed with PBS, and fresh isolated neutrophils or mononuclear cells were added as indicated (myofibroblasts previously incubated with neutrophils were incubated with mononuclear cells, and myofibroblasts incubated with mononuclear cells were incubated with neutrophils), and the AFM measurements were repeated.

#### 4.2.3. Protein–Protein Interaction Analysis

We aimed to assess the protein–protein interaction-based genome, which might be enriched in genes associated with cell phenotypes in the context of myocardial infarction. Protein–protein interaction analysis was performed using PPI networks from humans (*homo sapiens*). This analysis emphasized the known and predicted interactions of collagen proteins. The known interactions were defined from curated and experimental databases, whereas the predicted interactions included gene neighborhood, gene fusions and gene co-occurrence. The protein–protein interaction network of candidate genes was constructed in the STRING 11.0 (http://string-db.org, accessed on 20 November 2020) database, which is open-source software for the prediction and visualization of complex networks [79]. Targets with 28 kinds of collagen subtypes and representative protein of the cell phenotype (proliferation, apoptosis, inflammation, angiogenesis, remodeling, differentiation and migration) were selected to construct various functional clusters of the PPI network: anchoring, network forming, fibril-associated collagens with interrupted triple helices (FACIT), fibril, globular, multiplexing and transmembrane. A medium confidence score of 0.4 was set as the minimum required for the resulting network, which was analyzed with Cytoscape software (version 3.72). Each functional cluster includes nodes and edges that represent the target interactions between target collagen proteins and phenotype proteins, respectively.

#### 4.2.4. Statistical Analysis

Data represent mean ± SEM. Statistical analysis was performed with Prism5 software (GraphPad) using one-way ANOVA followed by Tukey’s post hoc test as indicated. When appropriate, a *t*-test or two-way ANOVA followed by Tukey’s post hoc test was performed. *p*-values < 0.05 were considered significant. Because most compliance AFM data, which are calculated based on force–distance deformation, present a bimodal distribution; data are fitted with a non-linear regression function, such as a double Lorentzian fitting function, using the constrained fitting amplitude. The widths were extracted and represented by mean ± SEM values over more than 15 height thumbnails per sample.

## Figures and Tables

**Table 1:** Influence of immune cells on signaling pathways in myofibroblasts.

Signaling Pathway	Hypoxia	Hypoxia + Neutrophils	Hypoxia + Mononuclear Cells
Collagen		↓ 1,3,5,16,23	↑ 1,3,23 ↓ 5,16
Remodeling	↑ TGFß1, IL1α ↑ AngII ↑ MMP1 ↓ MMP3, MMP9	↑ TGFß1 ↑ IL1, IL1ß	↑ AngII, NFkB
Migration	↑ IL1α	↑ IL1a, IL1ß	-
Proliferation	↑ PPARγ, PPARδ ↑ AT1R, AngII	↑ PPARγ	↑ PPARδ
Apoptosis			
Differentiation	↑ TGFß1	↑ TGFß1	-
Inflammation	↑ IL1α ↑ Syndecan4	↑ IL1, IL1ß ↑ Syndecan4	↓ Cadherin 11
Angiogenesis	↑ VEGFR1 ↑ CXCL12 ↓ Endothelin1	↑ VEGFR1	-

**Table 2:** Primers used to determine mRNA expression.

Collagen Type	Forward Primer	Reverse Primer
Collagen I	ACTACTGGAGAAGTTGGCAAGC	GTACCACGTTCTCCTCTTGGAC
Collagen II	GGGCAGACTGCAAAATAAAATC	GCGTCTGACTCACACCAGATAG
Collagen III	TCTGAGCTGCTTCTTCCTCTCT	GAAGAAACCAGGTTCCACTTTG
Collagen IV	CCTTCCTGCAGTCTTCCTAAAA	CAGTGAGCTCAGCTATCATTGG
Collagen V	CACTTACATCTGCCAGAACTCG	CTGGTTGAAGGACAACTCTTCC
Collagen VI	AGTGGCTAGTCCTTCCACTCTG	TGGATTGATTCTGACAGGACAC
Collagen VII	ACCTTGGGACCTGAGTCTAACA	AGGACAGTCAGCCATACTTGGT
Collagen VIII	ATGGATAGATGCCTGGAGTGAT	ATCTCCACAGATGACTGCTTCA
Collagen IX	CTTGTCCATGGAAGGAAGGTAG	TCAAACTCCTGGGAAGAGGATA
Collagen X	GGTTCATGGGATGTTTTATGCT	GGCGTATGGGATGAAGTATTGT
Collagen XI	CTGGTTTACCTGGTCTCAAAGG	GGAGGACCAATCAATCCAATTA
Collagen XII	TTCACAGTGGAGGATTTTGATG	TGCCAACTCTTGCTCAATTCTA
Collagen XIII	CTGCAGAAGAGAGAAGGGAAAG	ATCGGAGTACGCCAAAGTAAGA
Collagen XIV	AATTTGGTGGCTACAAACTGCT	CTTTTGTTGCAGTGTTCTGGAG
Collagen XV	GCTTTTTAACAATTGGGACTCG	ACCGTCAAAGGAGTAGATTGGA
Collagen XVI	TGGGAGACATGGTGAATTATGA	AGCCATCCTCTCATCAAACAGT
Collagen XVII	GACACCACACTAGACCCAGACA	CTTCCTCAGCATCAGGAGTTCT
Collagen XVIII	AAACTACTGGGGCTACAGGTCA	ACAGGACGATGTAGCTGTTGTG
Collagen XIX	CAAGGGAGAAATTGGTGAAAAG	TCAAACCATCTTGTCCATTGAG
Collagen XX	ACCGTCAGAGTCACCTGTTTTT	GCTAGGAGCTTTCTGTGTGGTT
Collagen XXII	CTCTATGAACAAGCTGGTGCTG	ATTGAAACCAGCATGAGGAACT
Collagen XXIII	GACGGCATTCCTGGACTAAAG	TGTCACCTTGTTCTCCCTTGAG
Collagen XXIV	GGACCTTAATCAGCCACAGTTC	AAGAGGATTCTTGATGCTGCTC
Collagen XXV	TATCAGAGCTCAGGTTGCTGAA	GACGACCTTTGGTAAAGTCTGC
Collagen XXVI	TGGCTGGTGCTTGGTCTC	CGAGGGTGGTGTTTTCTGTTC
Collagen XXVII	GTTGGAAATCCTAGAACGATGC	TCTGTCTGGTTTTCTTGGAGGT
Collagen XXVIII	GAAGACTCCAGGAAAGCAGTGT	AGAAGACAGAAGGCAACGTCTC
Biglycan	TGATTGAGAATGGGAGCCTGAG	CCTTGGTGATGTTGTTGGAGTG
Elastin	TCTTGCTGATCCTCTTGCTCA	GGATAATAGACTCCACCGGGAA
Fibronectin	GTGACACCCACCAGCTTTAC	ATCACCGATGAGCTGTCTGG
TGF-ß1	AGTGTGGAGCAACATGTGAAC	TTCAGCCACTGCCGTACAAC
Ang2	CGCTAACCAACCAAAGGCAC	TTACTGCTGAACTCCCACGG
AT1R	GGCAAGCAGAATAGGTGGGT	GCCACACCACTTCCTAACCA
PPARδ	GGGGGTCAGTCATGGAACAG	GTGTGTTCTGGTCCCCCATT
PPARγ	GACCGAGTGTGACGACAAGATT	AGCTGATTCCGAAGTTGGTGG
Syndecan4	CACCGAGGGTTAAGCTGGTT	ACCAGATGACAGGAGTCCCT
Cadherin11	GCCGACTTGTGAATGGGACT	GTAATTTCTGGGGCCGTTGC
IL-1α	TGCAAGCTATGGCTCACTTCA	CTTCCCGTTGCTTGACGTTG
IL-1ß	CAACCAACAAGTGATATTCTCCA	GATCCACACTCTCCAGCTGCA
Endothelin-1	CAGAAGTTGACGCACAACCG	TTGCTAAGATCCCAGCCAGC
VEGFR-1	TTCACCATCCCAAGGCAGTC	TGAGTCCTAGCTGGAGAGGG
NFkB	CCCTTATCGACCACCCCAAT	TGCTCCTGAGCATTGACTTCT
MMP1	GGAGGGGATACCCACTTTGA	ATGGCGGAGGGATCGTTAG
MMP3	GTTGGGCTTAAGAAGGTGGAC	GGACCGGAAGACCCTTCATT
MMP9	CAGACGTGGGTCGATTCCAA	TCATCGATCATGTCTCGCGG
p53	CTCCTTGGCTGTAGGTAGCG	TCCGACTGTGACTCCTCCAT
Bax	TGCAGAGGATGATTGCTGAC	GATCAGCTCGGGCACTTTAG
Bcl2	AGGAGCAGGTGCCTACAAGA	GCATTTTCCCACCACTGTCT
α-actin	GCATCCACGAAACCACCTA	CACGAGTAACAAATCAAAGC
β-actin	AGCCATGTACGTAGCCATCC	CTCTCAGCTGTGGTGGTGAA

## Data Availability

The data is available on request from the corresponding authors.

## Supplementary Materials

The following supporting information can be downloaded at: https://www.mdpi.com/article/10.3390/ijms232314571/s1.
