# Supplementary material for: Endogenous Modulation of Extracellular Matrix Collagen during Scar Formation after Myocardial Infarction
Source: Int J Mol Sci. 2022 Nov 23;23(23):14571. doi: 10.3390/ijms232314571 (PMC9741070; doi:10.3390/ijms232314571)
Supplement: Supplementary file 1 [file ijms-23-14571-s001.zip › ijms-1972131-supplementary.pdf]

Supplemental Table S1

Endogenous modulation of extracellular matrix collagen during scar formation after myocardial infarction

David Schumacher[1,2,3], MD, Adelina Curaj[3,4], MD, Mareike Staudt[3], Sakine Simsekyilmaz[3], Isabella Kanzler[3], PhD, Peter Boor[5,6,7], MD, PhD, Barbara Mara Klinkhammer[5], PhD, Xiofang Li[3], PhD, Octavian Bucur[8,9], MD, Adnan Kaabi[4], Yichen Xu[4,10], Huabo Zhang[4,10], Pakhwan Nilcham[4], Alexander Schuh[4], MD, PhD, Mihaela Rusu[4#*], PhD, Elisa A. Liehn[4,8,10#*], MD, PhD

[1]Department of Anesthesiology, University Hospital, RWTH Aachen University, 52074 Aachen, Germany.
[2] Institute of Experimental Medicine and Systems Biology, RWTH Aachen University, 52074 Aachen, Germany.
[3] Institute for Molecular Cardiovascular Research (IMCAR), RWTH Aachen University, 52074 Aachen, Germany.
[4] Department for Cardiology, Angiology and Internal Intensive Care, Medical Faculty, RWTH Aachen University, Germany
[5] Institute for Pathology, RWTH Aachen University, 52074 Aachen, Germany.
[6] Division of Nephrology and Clinical Immunology, RWTH Aachen University, Germany.
[7] Institute of Molecular Biomedicine, Comenius University, Bratislava, Slovakia
[8] National Institute of Pathology "Victor Babes", Bucharest, 050096 Bucharest, Romania
[9] Viron Molecular Medicine Institute, 1 Boston Place, Ste 2600, Boston, MA 02108, USA
[10] Institute for Molecular Medicine, University of Southern Denmark, 5230 Odense, Denmark

Supplemental Table S1. *Collagen subtypes: current state of art*

Type	Polymerrized form	Gene(s)	RefSeqGene	Tissue distribution	Pathways	Reference(s)
I	fibril	COL1A1	LRG_1 on chromosome 17	Internal organs, bones, teeth, tendons, ligaments, skin, cornea,	1. ECM-receptor interaction<br>2. Platelet activation	[1-3][22-26]

					3. Relaxin signaling pathway<br>4. Inflammatory Response Pathway<br>5.Focal adhesion<br>6. 3. PI3K-Akt signaling pathway<br>7. Protein digestion and absorption	
		COL1A2	LRG_2 on chromosome 7	Artery wall, endomysium of myofibrils, scar tissue	1.Cell surface interactions at the vascular wall<br>2. GPVI-mediated activation cascade<br>3. Platelet activation, signaling and agregation.<br>4. Relaxin signaling pathway<br>5. ECM-receptor interaction<br>6. Inflammatory Response Pathway<br>7.Focal adhesion<br>8. 3. PI3K-Akt signaling pathway	[1, 2],[27-29]

II	fibril	COL2A1	chromosome 12	cartilage, vitreous humour, notochord, intervertebral disc	1. integrin cell surface interactions<br>2.Signaling by PDGF<br>3.Signal transduction<br>4. Focal Adhesion<br>5. ECM-receptor interaction.<br>6. 3. PI3K-Akt signaling pathway	[1-3]
III	fibril	COL3A1	LRG_3 on chromosome 2	blood vessels, skin, lung, uterus, intestine, granulation tissue	1. integrin cell surface interactions<br>2.Inflammatory Response Pathway<br>3.Platelet Aggregation Inhibitor Pathway, Pharmacodynamics<br>4.NCAM1 interactions<br>5.Signaling by PDGF<br>6.Signal transduction<br>7. Protein digestion and absorption	[1, 2], [30,31]

IV	network-forming (integral components of basement membranes)	COL4A1<br>COL4A2	chromosome 13	all basal lamina, eye lens, capillaries, glomerular basement membranes	1.Focal Adhesion<br>2. ECM-receptor interaction<br>3. PI3K-Akt signaling pathway<br>4. Relaxin signaling pathway	[2, 3], [32-35]
		COL4A3<br>COL4A4	chromosome 2		1.Integrin cell surface interactions<br>2. Signaling by PDGF;<br>3.Signal transduction<br>4. ECM-receptor interaction<br>5. Focal Adhesion<br>6. Relaxin signaling pathway	
					1. ECM-receptor interaction<br>2. Focal Adhesion<br>3. Relaxin signaling pathway	
		COL4A5<br>COL4A6	chromosome X			

V	fibril (with type I)	COL5A1	chromosome 9 NG_008030.1	as type I, bone matrix, , placenta , corneal stroma, the interstitial matrix of muscles, liver, lungs	1.NCAM1 interactions<br>2.Signaling by PDGF<br>3. Signal transduction<br>4. regulating integrin-dependent activation of cardiac fibroblasts<br>5. Protein digestion and absorption	[1, 2], [36-38]
		COL5A2	chromosome 2 NG_011799.3		1.Focal adhesion<br>2. Protein digestion and absorption	
		COL5A3	Chromosome 19 NG_046943.1		1. Focal adhesion<br>2. Protein digestion and absorption	
VI	beaded filament collagen	COL6A1	chromosome 21 (21q22.3)	as type I, interstitial tissue, skeletal muscle, lung, kidney, cornea, tendon, dermis, cartilage, intervertebral discs, blood vessels, adipose tissue.<br><br>Interface between the basement membrane and interstitial matrix	1.NCAM1 interactions<br>2. Signaling by PDGF<br>3. Signal transduction<br>4. Focal adhesion<br>5. ECM-receptor interaction<br>6. PI3K-Akt signaling pathway<br>7. Protein digestion and absorption<br><br>*Col6a1-may promote	[39-41]

		COL6A2	Chormosome21 (21q22.3)		macrophage migration and polarization via AKT and PKA pathways	
		COL6A3	chromosome 2 (2q37)		1.ECM receptor interaction<br>2. Focal adhesion<br>3. PI3K-Akt signaling pathway<br>4. Protein digestion and absorption	
		COL6A4 (The α4 chain is absent in humans)	pseudogenes due to a chromosome break			
		COL6A5	chromosome 3 (3q22.1)		1.ECM receptor interaction<br>2. Focal adhesion<br>3. PI3K-Akt signaling pathway<br>4. Protein digestion and absorption	
		COL6A6	Chormosome3 (3q22.1)	α6 chain -rich in the endomysium, perimysium of skeletal muscle, myocardium	1.ECM receptor interaction<br>2. Focal adhesion<br>3. PI3K-Akt signaling pathway	

VII	anchoring fibrils	COL7A1	chromosome 3	rectum, colon, small intestine, placenta, esophagus, oral mucosa, cervix, skeletal muscle, skin, cornea<br>-forms anchoring fibrils beneath stratified squamous epithelia	- Involve in stability of interstitial membrane (binds to type I and type III collagen), basement membrane ECM structures.	[4],[42]
VIII	network-forming	COL8A1	Chormosome3 (3q11.1-q13.2)	bone, cartilage, brain, eye, kidney, heart, vascular tissues, liver, lung, muscle, skin, spleen, nerves, ligaments and tendons,	Involve in angiogenesis, tissue remodeling, fibrosis<br><br>Col8a1- promotes smooth muscle cells proliferation and migration	[5],[43,44]
	nonfibrilar	COL8A2	chromosome 1 (1p34.2-p32.3)			
IX	fibril-associated collagen with interrupted triple helices (FACITs)	COL9A1	chromosome 6	cartilage with type II and XI fibrils; chondrocytes of growth-plate cartilage, articular cartilage in adult, inner ear, intervertebral discs	1.ECM receptor interaction<br>2. Focal adhesion<br>3. PI3K-Akt signaling pathway	[1, 2], [41,45]

				-form the unique heterofibril network in the matrix of cartilage by combine with types II and XI collagen		
		COL9A2	chromosome 1		1.NCAM1 interactions<br>2.Signaling by PDGF<br>3.Signal transduction<br>4. ECM receptor interaction<br>5. Focal adhesion<br>6. PI3K-Akt signaling pathway	
		COL9A3	chromosome 20		1.ECM receptor interaction<br>2. Focal adhesion<br>3. PI3K-Akt signaling pathway	
X	network-forming nonfibrillar collagen	COL10A1	chromosome 6	growth plate (hypertrophic zone) and articular cartilage (basal calcified zone)	1.Endochondral Ossification<br>2.Mineralization<br>3. Protein digestion and absorption	[2],[46,47]
XI	fibril (with type II)	COL11A1<br><br>COL11A2	chromosome 1<br><br>chromosome 6	As for type II, skeletal muscle, articular cartilage, intervertebral discs, tendons, trachea,	1.ECM–receptor interaction<br>2.focal adhesion	[48-50]

		COL11A3	chromosome12	trabecular bone, placenta, testis, lung, neoepithelium of brain	(1,2) Invlove in cancer delvelopement<br>3. Protein digestion and absorption<br><br>-involve fibrillogenesis (nucleator for the of type I and II collagen)<br>- inhibit matrix degradation by reducing the release of GAGs in human articular chondrocyte pellets	
XII	fibril-associated collagen with interrupted triple helices (FACIT)	COL12A1	Chromosome 6	Associate with type I collagen, bones, dense conenctive tissue, interstitial matrix, component of skeletal muscle	Protein digestion and absorption<br><br>- temporarily stabilize type I collagen fibrils, prevents the fibrils from permanently crosslinking	[6],[41,51,52]
XIII	nonfibrillar, type II trans-membrane collagen	COL13A1	Chromosome 10	connective tissues; blood vessels, bone, neuromuscular structures	1.cell adhesion<br>2.adhesion-dependent cell functions	[7, 8],[53]
XIV	fibril-associated collagen with	COL14A1	Chromosome 8	skin, cornea, articular cartilage, tendon	Fibrillogenesis	[54-55]

	interrupted triple helices (FACIT)					
XV	endostatin precursors collagens	COL15A1	Chromosome 9	cardiac skeletal myocytes, basement membrane of microvessels, small intestine, colon, kidney, pancreas, testes, ovaries, prostate	1.Extracellular matrix organization<br>2.Assembly of collagen fibrils and other multimeric structures<br>3. Protein digestion and absorption	[3],[56-58]
XVI	fibril-associated collagens with interrupted triple helices (FACITs)	COL16A1	Chromosome 1	heart, arterial walls, intestine, kidney, skin, cartilage	1.Focal adhesion<br>2.Integrin mediated signaling pathway<br>3.extracellular metrix organization<br>4. Protein digestion and absorption	[9],[59-62]
XVII	non-fibrillar/ type II transmembrane collagen	COL17A1	Chromosome 10	Skin (epithelial hemides-mosomes), colonic mucosa, brain, kidney, placenta, cornea	1.Type I hemidesmosome assembly<br>2.Cell junction organization<br>3.Cell-cell comunication<br>4.a6b1 and a6b4 Integrin signaling<br>5. Protein digestion and absorption	[3],[63-64]

XVIII	Multiplexin ;Multiple Triple Helix domains with Interruptions	COL18A1	Chromosome 21	fat tissue (during adipose differentiation), basement membrane, eye, articular cartilage, bone marrow, hair follicles , heart, plasma, brain, liver, kidneys	1.Extracellular matrix organization<br>2.inhibition of angiogenesis<br>3.inhibition of Wnt/β-catenin signaling and development of the eye<br>4.Protein digestion and absorption<br><br>-Endostatin (carboxyl-terminal fragment of type XVIII collagen) bind receptor KDR/Flk-1 interfering with VEGF signaling lead to inhibition of proliferation and migration of endothelial cell.<br><br>- modulate PDGFR/Erk pathway; reducing TGF-β	[3],[65-68]
XIX	member of the fibril-associated	COL19A1	Chromosome 6 (6q12q14)	breast (basement membrane zones), kidney, liver, colon,	1. extracellular matrix organization	[10, 11],[69-70]

	collagens with interrupted triple helices (FACIT)			placenta, spleen ,prostate, skeletal muscle, skin	2.Protein digestion and absorption	
XX	fibril-associated collagens with interrupted triple helices (FACIT)	COL20A1	Chromosome 20 (20q13.33)	corneal epithelium, tendon, embryonic skin, sternal cartilage	1.Cellular adhesion<br>2. Cells migration,differentiation, and signaling<br>3. Protein digestion and absorption	[12],[41, 71]
XXI	fibril-associated collagens with interrupted triple helices (FACIT)	COL21A1	Chromosome 6	heart, lung, kidney, placenta, jejunum, stomach, skeletal muscle, lympnode, pancreas	1.Protein digestion and absorption<br>2. maintains integrity of extracellular matrix (localized with type I collagen)	[11,13],[41,72-73]
XXII	fibril-associated collagens with interrupted triple helices (FACIT)	COL22A1	Chromosome 8	heart, cartilage and skin	1.The sequences GLQGER and GFKGER are binding motifs between collagen XXII and α2β1 integrin<br>2. Cell adhesion<br>3. integrates the junctional extracellular matrix	[14,15],[41,74-75]

XXIII	type II trans-membrane collagen	COL23A1	Chromosome 5	cornea, lung, skin, tendon, amnion, placenta, kidney	may involve in formation or maintenance of cell–cell contacts or epithelial cells polarization	[76-77]
XXIV	fibrillar collagen	COL24A1	Chromosome1 (1p.22.3)	Bone (predominantly), brain, muscle, kidneys, liver, spleen, lung, testis, ovary	1. Involve in collagen fibril organization<br>2. Involve in ECM organization<br><br>-known as marker of osteoblast differentiation and bone formation	[16],[41,78]
XXV	membrane-associated collagens with interrupted triple helices (MACITs)	COL25A1	Chromosome 4	neurons of the brain (predominantly), heart, testis, retina<br><br>senile plaques of Alzheimer's disease	1. involved in cell–cell, cell–matrix adhesion<br><br>2.involved in fibrillization and cell toxicity<br><br>3. potential play role in Alzheimer's-disease (protects against proteolysis of amyloid β-peptides)	[17],[41,79-80]

XXVI	does not fit within any of the collagen family subgroups but contained collagenous domains	COL26A1 (EMID2)	Chromosome 7	testis, ovary	-may involve as extracellular matrix component of early development of testis and ovary	[18],[81-83]
XXVII	third class (type C) fibrillar collagen	COL27A1	Chromosome 9 (9q32)	Cartilage in adult, epiphyseal growth plate in children and adolescents.<br><br>During fetal development; in major arteries of the heart, endochondral bone, lungs, ear, colon, retina, cornea	structural role in the pericellular extracellular matrix of the growth plate, organisation of the proliferative zone	[19, 20],[84]
XXVIII	collagens containing von Willebrand factor	COL28A1	Chromosome 7 (7p21.3)	peripheral nerves, glial cells, dorsal root ganglia, nodes of Ranvier	1. Cell adhesion<br>2. ECM organization<br><br>-may involve in repair process	[21],[85-87]

1. in *Molecular Biology of the Cell.*
2. Koide, T., *Designed triple-helical peptides as tools for collagen biochemistry and matrix engineering.* Philos Trans R Soc Lond B Biol Sci, 2007. **362**(1484): p. 1281-91.
3. Gordon, M.K. and R.A. Hahn, *Collagens.* Cell Tissue Res, 2010. **339**(1): p. 247-57.
4. Iinuma, S., et al., *Transplanted bone marrow-derived circulating PDGFRalpha+ cells restore type VII collagen in recessive dystrophic epidermolysis bullosa mouse skin graft.* J Immunol, 2015. **194**(4): p. 1996-2003.
5. Skrbic, B., et al., *Lack of collagen VIII reduces fibrosis and promotes early mortality and cardiac dilatation in pressure overload in mice.* Cardiovasc Res, 2015. **106**(1): p. 32-42.
6. Chiquet, M., et al., *Collagen XII: Protecting bone and muscle integrity by organizing collagen fibrils.* Int J Biochem Cell Biol, 2014. **53**: p. 51-4.
7. Ylonen, R., et al., *Type XIII collagen strongly affects bone formation in transgenic mice.* J Bone Miner Res, 2005. **20**(8): p. 1381-93.
8. Vaisanen, M.R., T. Vaisanen, and T. Pihlajaniemi, *The shed ectodomain of type XIII collagen affects cell behaviour in a matrix-dependent manner.* Biochem J, 2004. **380**(Pt 3): p. 685-93.
9. Grassel, S. and R.J. Bauer, *Collagen XVI in health and disease.* Matrix Biol, 2013. **32**(2): p. 64-73.
10. Amenta, P.S., et al., *Loss of types XV and XIX collagen precedes basement membrane invasion in ductal carcinoma of the female breast.* J Pathol, 2003. **199**(3): p. 298-308.
11. Tuckwell, D., *Identification and analysis of collagen alpha 1(XXI), a novel member of the FACIT collagen family.* Matrix Biol, 2002. **21**(1): p. 63-6.
12. Koch, M., et al., *alpha 1(Xx) collagen, a new member of the collagen subfamily, fibril-associated collagens with interrupted triple helices.* J Biol Chem, 2001. **276**(25): p. 23120-6.
13. Fitzgerald, J. and J.F. Bateman, *A new FACIT of the collagen family: COL21A1.* FEBS Lett, 2001. **505**(2): p. 275-80.
14. Zwolanek, D., et al., *Collagen XXII binds to collagen-binding integrins via the novel motifs GLQGER and GFKGER.* Biochem J, 2014. **459**(1): p. 217-27.
15. Koch, M., et al., *A novel marker of tissue junctions, collagen XXII.* J Biol Chem, 2004. **279**(21): p. 22514-21.
16. Wang, W., et al., *Collagen XXIV (Col24alpha1) promotes osteoblastic differentiation and mineralization through TGF-beta/Smads signaling pathway.* Int J Biol Sci, 2012. **8**(10): p. 1310-22.
17. Tong, Y., et al., *COL25A1 triggers and promotes Alzheimer's disease-like pathology in vivo.* Neurogenetics, 2010. **11**(1): p. 41-52.

18. Sato, K., et al., *Type XXVI collagen, a new member of the collagen family, is specifically expressed in the testis and ovary.* J Biol Chem, 2002. **277**(40): p. 37678-84.
19. Plumb, D.A., et al., *Collagen XXVII organises the pericellular matrix in the growth plate.* PLoS One, 2011. **6**(12): p. e29422.
20. Hjorten, R., et al., *Type XXVII collagen at the transition of cartilage to bone during skeletogenesis.* Bone, 2007. **41**(4): p. 535-42.
21. Veit, G., et al., *Collagen XXVIII, a novel von Willebrand factor A domain-containing protein with many imperfections in the collagenous domain.* J Biol Chem, 2006. **281**(6): p. 3494-504.
22. Santiago, J.-J. *et al.* Cardiac fibroblast to myofibroblast differentiation in vivo and in vitro: Expression of focal adhesion components in neonatal and adult rat ventricular myofibroblasts. *Developmental Dynamics* **239**, 1573–1584 (2010).
23. COL1A1 Gene - GeneCards | CO1A1 Protein | CO1A1 Antibody. https://www.genecards.org/cgi-bin/carddisp.pl?gene=COL1A1&keywords=COL1A1.
24. Xu, J.-Y., Xiong, Y.-Y., Lu, X.-T. & Yang, Y.-J. Regulation of Type 2 Immunity in Myocardial Infarction. *Front. Immunol.* **10**, (2019)
25. Ruan, Y. *et al.* Grape Seed Proanthocyanidin Extract Ameliorates Cardiac Remodelling After Myocardial Infarction Through PI3K/AKT Pathway in Mice. *Front Pharmacol* **11**, 585984 (2020).
26. Zhao, L., Yang, X., Rong & Han, X. MicroRNA-146b induces the PI3K/Akt/NF-κB signaling pathway to reduce vascular inflammation and apoptosis in myocardial infarction by targeting PTEN. *Exp Ther Med* **17**, 1171–1181 (2019).
27. COL1A2 Gene - GeneCards | CO1A2 Protein | CO1A2 Antibody. https://www.genecards.org/cgi-bin/carddisp.pl?gene=COL1A2&keywords=col1a2.
28. COL2A1 collagen type II alpha 1 chain [Homo sapiens (human)] - Gene - NCBI. https://www.ncbi.nlm.nih.gov/gene/1280.
29. Israeli-Rosenberg, S., Manso, A. M., Okada, H. & Ross, R. S. Integrins and Integrin-Associated Proteins in the Cardiac Myocyte. *Circ Res* **114**, 572–586 (2014).
30. COL3A1 collagen type III alpha 1 chain [Homo sapiens (human)] - Gene - NCBI. https://www.ncbi.nlm.nih.gov/gene/1281.
31. Mandic, L. et al. Molecular Imaging of Angiogenesis in Cardiac Regeneration. Curr Cardiovasc Imaging Rep 9, 27 (2016).
32. Sand, J. M. B., Genovese, F., Gudmann, N. S. & Karsdal, M. A. Chapter 4 - Type IV collagen. in *Biochemistry of Collagens, Laminins and Elastin (Second Edition)* (ed. Karsdal, M. A.) 37–49 (Academic Press, 2019). doi:10.1016/B978-0-12-817068-7.00004-5.
33. COL4A1 Gene - GeneCards | CO4A1 Protein | CO4A1 Antibody. https://www.genecards.org/cgi-bin/carddisp.pl?gene=COL4A1&keywords=col4a1.
34. COL4A1 collagen type IV alpha 1 chain [Homo sapiens (human)] - Gene - NCBI. https://www.ncbi.nlm.nih.gov/gene/1282.
35. COL4A2 collagen type IV alpha 2 chain [Homo sapiens (human)] - Gene - NCBI. https://www.ncbi.nlm.nih.gov/gene/1284.

36. Cho, S., Paik, D. T. & Wu, J. C. An extracellular matrix paradox in myocardial scar formation. *Signal Transduct Target Ther* **5**, (2020).
37. Leeming, D. J. & Karsdal, M. A. Chapter 5 - Type V collagen. in *Biochemistry of Collagens, Laminins and Elastin (Second Edition)* (ed. Karsdal, M. A.) 51–57 (Academic Press, 2019). doi:10.1016/B978-0-12-817068-7.00005-7.
38. COL5A3 collagen type V alpha 3 chain [Homo sapiens (human)] - Gene - NCBI. https://www.ncbi.nlm.nih.gov/gene/50509
39. Sun, S., Genovese, F. & Karsdal, M. A. Chapter 6 - Type VI collagen. in *Biochemistry of Collagens, Laminins and Elastin (Second Edition)* (ed. Karsdal, M. A.) 59–67 (Academic Press, 2019). doi:10.1016/B978-0-12-817068-7.00006-9.
40. Liu, J. *et al.* Proteomics Profiling Reveals Insulin-Like Growth Factor 1, Collagen Type VI α-2 Chain, and Fermitin Family Homolog 3 as Potential Biomarkers of Plaque Erosion in ST-Segment Elevated Myocardial Infarction. *Circ J* **84**, 985–993 (2020).
41. KEGG GENES Database. https://www.genome.jp/kegg/genes.html.
42. Mortensen, J. H. & Karsdal, M. A. Chapter 7 - Type VII collagen. in Biochemistry of Collagens, Laminins and Elastin (Second Edition) (ed. Karsdal, M. A.) 69–74 (Academic Press, 2019). doi:10.1016/B978-0-12-817068-7.00007-0.
43. COL8A1 collagen type VIII alpha 1 chain [Homo sapiens (human)] - Gene - NCBI. https://www.ncbi.nlm.nih.gov/gene/1295.
44. Gil-Cayuela, C. et al. New Altered Non-Fibrillar Collagens in Human Dilated Cardiomyopathy: Role in the Remodeling Process. PLoS One 11, (2016).
45. He, Y., Sardar, S. & Karsdal, M. A. Chapter 9 - Type IX collagen. in Biochemistry of Collagens, Laminins and Elastin (Second Edition) (ed. Karsdal, M. A.) 83–89 (Academic Press, 2019). doi:10.1016/B978-0-12-817068-7.00009-4.
46. He, Y., Gudmann, N. S., Willumsen, N. & Karsdal, M. A. Chapter 10 - Type X collagen. in Biochemistry of Collagens, Laminins and Elastin (Second Edition) (ed. Karsdal, M. A.) 91–97 (Academic Press, 2019). doi:10.1016/B978-0-12-817068-7.00010-0.
47. COL10A1 collagen type X alpha 1 chain [Homo sapiens (human)] - Gene - NCBI. https://www.ncbi.nlm.nih.gov/gene/1300.
48. Luo, Y. Y., Szlarski, P. M., Kehlet, S. N. & Karsdal, M. A. Chapter 11 - Type XI collagen. in Biochemistry of Collagens, Laminins and Elastin (Second Edition) (ed. Karsdal, M. A.) 99–106 (Academic Press, 2019). doi:10.1016/B978-0-12-817068-7.00011-2.

49. Li, A., Wei, Y., Hung, C. & Vunjak-Novakovic, G. Chondrogenic properties of collagen type XI, a component of cartilage extracellular matrix. Biomaterials 173, 47–57 (2018).
50. COL11A1 collagen type XI alpha 1 chain [Homo sapiens (human)] - Gene - NCBI. https://www.ncbi.nlm.nih.gov/gene/1301.
51. Mortensen, J. H., Manon-Jensen, T. & Karsdal, M. A. Chapter 12 - Type XII collagen. in Biochemistry of Collagens, Laminins and Elastin (Second Edition) (ed. Karsdal, M. A.) 107–113 (Academic Press, 2019). doi:10.1016/B978-0-12-817068-7.00012-4.
52. COL12A1 collagen type XII alpha 1 chain [Homo sapiens (human)] - Gene - NCBI. https://www.ncbi.nlm.nih.gov/gene/1303.

53. Siebuhr, A. S., Thudium, C. S. & Karsdal, M. A. Chapter 13 - Type XIII collagen. in Biochemistry of Collagens, Laminins and Elastin (Second Edition) (ed. Karsdal, M. A.) 115–120 (Academic Press, 2019). doi:10.1016/B978-0-12-817068-7.00013-6.
54. COL14A1 collagen type XIV alpha 1 chain [Homo sapiens (human)] - Gene - NCBI. https://www.ncbi.nlm.nih.gov/gene/7373.
55. Freise, C., Bobb, V. & Querfeld, U. Collagen XIV and a related recombinant fragment protect human vascular smooth muscle cells from calcium-/phosphate-induced osteochondrocytic transdifferentiation. Experimental Cell Research 358, 242–252 (2017).
56. Manon-Jensen, T., Arvanitidis, A. & Karsdal, M. A. Chapter 15 - Type XV collagen. in Biochemistry of Collagens, Laminins and Elastin (Second Edition) (ed. Karsdal, M. A.) 127–131 (Academic Press, 2019). doi:10.1016/B978-0-12-817068-7.00015-X.
57. COL15A1 collagen type XV alpha 1 chain [Homo sapiens (human)] - Gene - NCBI. https://www.ncbi.nlm.nih.gov/gene/1306.
58. PB | Extracellular matrix organization. https://reactome.org/PathwayBrowser/#/R-HSA-1474244.
59. Sand, J. M. B., Jensen, C. & Karsdal, M. A. Chapter 16 - Type XVI collagen. in Biochemistry of Collagens, Laminins and Elastin (Second Edition) (ed. Karsdal, M. A.) 133–139 (Academic Press, 2019). doi:10.1016/B978-0-12-817068-7.00016-1.
60. COL16A1 collagen type XVI alpha 1 chain [Homo sapiens (human)] - Gene - NCBI. https://www.ncbi.nlm.nih.gov/gene/1307.
61. Eble, J. A. et al. Collagen XVI harbors an integrin alpha1 beta1 recognition site in its C-terminal domains. J Biol Chem 281, 25745–25756 (2006).
62. Gaudet, P., Livstone, M. S., Lewis, S. E. & Thomas, P. D. Phylogenetic-based propagation of functional annotations within the Gene Ontology consortium. Brief Bioinform 12, 449–462 (2011).
63. Sun, S. & Karsdal, M. A. Chapter 17 - Type XVII collagen. in Biochemistry of Collagens, Laminins and Elastin (Second Edition) (ed. Karsdal, M. A.) 141–147 (Academic Press, 2019). doi:10.1016/B978-0-12-817068-7.00017-3.
64. COL17A1 collagen type XVII alpha 1 chain [Homo sapiens (human)] - Gene - NCBI. https://www.ncbi.nlm.nih.gov/gene/1308.
65. Yamaguchi, Y. et al. A Peptide Derived from Endostatin Ameliorates Organ Fibrosis. Science Translational Medicine 4, 136ra71-136ra71 (2012).
66. Pehrsson, M., Bager, C. L. & Karsdal, M. A. Chapter 18 - Type XVIII collagen. in Biochemistry of Collagens, Laminins and Elastin (Second Edition) (ed. Karsdal, M. A.) 149–162 (Academic Press, 2019). doi:10.1016/B978-0-12-817068-7.00018-5.
67. COL18A1 collagen type XVIII alpha 1 chain [Homo sapiens (human)] - Gene - NCBI. https://www.ncbi.nlm.nih.gov/gene/80781.
68. Li, Y. & Ren, H. Endostatin inhibits fibrosis by modulating the PDGFR/ERK signal pathway: an in vitro study. J Zhejiang Univ Sci B 18, 994–1001 (2017).
69. Nielsen, S. H. & Karsdal, M. A. Chapter 19 - Type XIX collagen. in Biochemistry of Collagens, Laminins and Elastin (Second Edition) (ed. Karsdal, M. A.) 163–166 (Academic Press, 2019). doi:10.1016/B978-0-12-817068-7.00019-7.

70. COL19A1 collagen type XIX alpha 1 chain [Homo sapiens (human)] - Gene - NCBI. https://www.ncbi.nlm.nih.gov/gene/1310.
71. Willumsen, N., Nissen, N. I. & Karsdal, M. A. Chapter 20 - Type XX collagen. in Biochemistry of Collagens, Laminins and Elastin (Second Edition) (ed. Karsdal, M. A.) 167–170 (Academic Press, 2019). doi:10.1016/B978-0-12-817068-7.00020-3.
72. Kehlet, S. N., Jessen, H. & Karsdal, M. A. Chapter 21 - Type XXI collagen. in Biochemistry of Collagens, Laminins and Elastin (Second Edition) (ed. Karsdal, M. A.) 171–174 (Academic Press, 2019). doi:10.1016/B978-0-12-817068-7.00021-5.
73. COL21A1 collagen type XXI alpha 1 chain [Homo sapiens (human)] - Gene - NCBI. https://www.ncbi.nlm.nih.gov/gene/81578.
74. Kehlet, S. N., Jessen, H. & Karsdal, M. A. Chapter 22 - Type XXII collagen. in Biochemistry of Collagens, Laminins and Elastin (Second Edition) (ed. Karsdal, M. A.) 175–179 (Academic Press, 2019). doi:10.1016/B978-0-12-817068-7.00022-7.
75. Knockdown of col22a1 gene in zebrafish induces a muscular dystrophy by disruption of the myotendinous junction | Development | The Company of Biologists. https://journals.biologists.com/dev/article/140/22/4602/45937/Knockdown-of-col22a1-gene-in-zebrafish-induces-a
76. Kehlet, S. N., Jessen, H. & Karsdal, M. A. Chapter 23 - Type XXIII collagen. in Biochemistry of Collagens, Laminins and Elastin (Second Edition) (ed. Karsdal, M. A.) 181–185 (Academic Press, 2019). doi:10.1016/B978-0-12-817068-7.00023-9.
77. COL23A1 collagen type XXIII alpha 1 chain [Homo sapiens (human)] - Gene - NCBI. https://www.ncbi.nlm.nih.gov/gene/91522.
78. COL24A1 collagen type XXIV alpha 1 chain [Homo sapiens (human)] - Gene - NCBI. https://www.ncbi.nlm.nih.gov/gene/255631.
79. Langholm, L. L., Kjeld, N. G. & Karsdal, M. A. Chapter 25 - Type XXV collagen. in Biochemistry of Collagens, Laminins and Elastin (Second Edition) (ed. Karsdal, M. A.) 191–196 (Academic Press, 2019). doi:10.1016/B978-0-12-817068-7.00025-2.
80. COL25A1 collagen type XXV alpha 1 chain [Homo sapiens (human)] - Gene - NCBI. https://www.ncbi.nlm.nih.gov/gene/84570.
81. Manon-Jensen, T., Kjeld, N. G. & Karsdal, M. A. Chapter 26 - Type XXVI collagen. in Biochemistry of Collagens, Laminins and Elastin (Second Edition) (ed. Karsdal, M. A.) 197–200 (Academic Press, 2019). doi:10.1016/B978-0-12-817068-7.00026-4.
82. COL26A1 collagen type XXVI alpha 1 chain [Homo sapiens (human)] - Gene - NCBI. https://www.ncbi.nlm.nih.gov/gene/136227.
83. Sato, K. et al. Type XXVI collagen, a new member of the collagen family, is specifically expressed in the testis and ovary. J Biol Chem 277, 37678–37684 (2002).
84. Genovese, F. & Karsdal, M. A. Chapter 27 - Type XXVII collagen. in Biochemistry of Collagens, Laminins and Elastin (Second Edition) (ed. Karsdal, M. A.) 201–204 (Academic Press, 2019). doi:10.1016/B978-0-12-817068-7.00027-6.
85. Sparding, N., Arvanitidis, A. & Karsdal, M. A. Chapter 28 - Type XXVIII collagen. in Biochemistry of Collagens, Laminins and Elastin (Second Edition) (ed. Karsdal, M. A.) 205–208 (Academic Press, 2019). doi:10.1016/B978-0-12-817068-7.00028-8.
86. COL28A1 collagen type XXVIII alpha 1 chain [Homo sapiens (human)] - Gene - NCBI. https://www.ncbi.nlm.nih.gov/gene/340267.

87. Schiller, H. B. et al. Time- and compartment-resolved proteome profiling of the extracellular niche in lung injury and repair. Mol Syst Biol 11, 819 (2015).
